# Clinical Studies and Pre-clinical Animal Models on Facial Nerve Preservation, Reconstruction, and Regeneration Following Cerebellopontine Angle Tumor Surgery–A Systematic Review and Future Perspectives

**DOI:** 10.3389/fbioe.2021.659413

**Published:** 2021-06-18

**Authors:** Isabel C. Hostettler, Narayan Jayashankar, Christos Bikis, Stefan Wanderer, Edin Nevzati, Ravindran Karuppiah, Vicknes Waran, Daniel Kalbermatten, Luigi Mariani, Serge Marbacher, Raphael Guzman, Srinivas Madduri, Michel Roethlisberger

**Affiliations:** ^1^Department of Neurosurgery, Klinikum Rechts der Isar, Technical University Munich, Munich, Germany; ^2^Department of Oto-Rhino-Laryngology, Nanavati Super Speciality Hospital, Mumbai, India; ^3^Department of Biomedical Engineering, Biomaterials Science Center, University of Basel, Allschwil, Switzerland; ^4^Integrierte Psychiatrie Winterthur - Zürcher Unterland, Winterthur, Switzerland; ^5^Department of Neurosurgery, Kantonsspital Aarau, Aarau, Switzerland; ^6^Department of Neurosurgery, Kantonsspital Luzern, Lucerne, Switzerland; ^7^Department of Neurosurgery, University Malaya Specialist Centre, University of Malaya, Kuala Lumpur, Malaysia; ^8^Department of Plastic Surgery, University Hospital Geneva, Geneva, Switzerland; ^9^Department of Surgery, Biomaterials and Neuro Tissue Bioengineering, University of Geneva, Geneva, Switzerland; ^10^Department of Neurosurgery, University Hospital of Basel, University of Basel, Basel, Switzerland; ^11^Department of Biomedicine, Brain Ischemia and Regeneration, University of Basel, Basel, Switzerland; ^12^Department of Biomedical Engineering, Center for Bioengineering and Regenerative Medicine, University of Basel, Basel, Switzerland

**Keywords:** cerebellopontine angle, facial nerve regeneration, vestibular schwannoma, meningioma, retrosigmoid approach, middle cranial fossa, nerve injury model, bioactive nerve conduits

## Abstract

**Background and purpose:** Tumorous lesions developing in the cerebellopontine angle (CPA) get into close contact with the 1st (cisternal) and 2nd (meatal) intra-arachnoidal portion of the facial nerve (FN). When surgical damage occurs, commonly known reconstruction strategies are often associated with poor functional recovery. This article aims to provide a systematic overview for translational research by establishing the current evidence on available clinical studies and experimental models reporting on intracranial FN injury.

**Methods:** A systematic literature search of several databases (PubMed, EMBASE, Medline) was performed prior to July 2020. Suitable articles were selected based on predefined eligibility criteria following the Preferred Reporting Items for Systematic Reviews and Meta Analyses (PRISMA) guidelines. Included clinical studies were reviewed and categorized according to the pathology and surgical resection strategy, and experimental studies according to the animal. For anatomical study purposes, perfusion-fixed adult New Zealand white rabbits were used for radiological high-resolution imaging and anatomical dissection of the CPA and periotic skull base.

**Results:** One hundred forty four out of 166 included publications were clinical studies reporting on FN outcomes after CPA-tumor surgery in 19,136 patients. During CPA-tumor surgery, the specific vulnerability of the intracranial FN to stretching and compression more likely leads to neurapraxia or axonotmesis than neurotmesis. Severe FN palsy was reported in 7 to 15 % after vestibular schwannoma surgery, and 6% following the resection of CPA-meningioma. Twenty-two papers reported on experimental studies, out of which only 6 specifically used intracranial FN injury in a rodent (*n* = 4) or non-rodent model (*n* = 2). Rats and rabbits offer a feasible model for manipulation of the FN in the CPA, the latter was further confirmed in our study covering the radiological and anatomical analysis of perfusion fixed periotic bones.

**Conclusion:** The particular anatomical and physiological features of the intracranial FN warrant a distinguishment of experimental models for intracranial FN injuries. New Zealand White rabbits might be a very cost-effective and valuable option to test new experimental approaches for intracranial FN regeneration. Flexible and bioactive biomaterials, commonly used in skull base surgery, endowed with trophic and topographical functions, should address the specific needs of intracranial FN injuries.

## Introduction

The human facial nerve (FN) contains an average of 7,500 and up to 9,370 somatomotoric axons. Between 3,120 and 5,360 somatosensory and secretomotory axons, are separately bundled within the intermedius (Wrisberg's) nerve, which is in close contact but without axonal exchange with the FN (Thurner et al., 1993). The axonal bundles are surrounded by a multilayered myelin sheath with a transitional zone between the central myelin generated by oligodendrocytes and the peripheral myelin produced by Schwann cells in a dome-shaped transitional zone. This so called Obersteiner-Redlich zone of the FN is found very close to the root exit zone at the brainstem, from a proximal to distal direction (Guclu et al., 2009). The 1st (cisternal) intra-arachnoidal segment, originating at the recess between the olive and the inferior peduncle of the brainstem to the internal acoustic porus (IAP), is the longest of the intracranial segments with an average length of 19.5 mm in humans (Captier et al., 2005). The 2nd (meatal) intra-arachnoidal segment extends from the IAP to the fundus of the internal acoustic canal (IAC). Within the 3rd (labyrinthine) segment, being the shortest in humans, the FN occupies a small rostro-dorsal portion of the IAC, and extends from the fundus of the IAC to the geniculate ganglion (Lescanne et al., 2002; Bendella et al., 2016). Within these first three segments, the neuronal axons are individually myelinated. The axonal bundles are surrounded by endoneurium and form fascicles, which are disorderly grouped and lack an undular patterning, known from extracranial nerves (Podvinec and Pfaltz, 1976; Sekiya et al., 1985; Ishii and Takeuchi, 1993; Captier et al., 2005). The 1st and 2nd/3rd segments of the FN are covered by a sheath of arachnoid membrane and lack a peri- and epineural layer (Podvinec and Pfaltz, 1976; Sekiya et al., 1985; Lescanne et al., 2002; Captier et al., 2005). Within the facial (Fallopian) canal of the petrous bone, the 4th (tympanic) and the 5th (mastoid) segment of the FN undergoes further divisions into the greater petrosal nerve and the chorda tympani after the geniculate ganglion junction. The entire nerve is surrounded by a perineurium and embedded within a protective epineurium, eventually ending up in the 6th (extratemporal) segment (Sekiya et al., 1985; Captier et al., 2005). The reduced protective layering of the intra-arachnoidal FN with a relative lack of direct stabilizing contacts to the dura mater may be sufficient in a physiological state, where this part of the nerve is not exposed to physical forces. Protection mostly occurs via the cerebrospinal fluid (CSF), the arachnoid layer and the cisternal anatomy. However, even in a physiological state, the intra-arachnoidal FN is strained and exposed to pulsatile forces within the subarachnoid space (Lescanne et al., 2002; Bendella et al., 2016).

Tumorous lesions developing in the CPA usually display a benign and slow growth pattern. They chronically affect the FN in its cisternal and meatal intracranial portion by dislocation, stretching, flattening, compression, and even engulfement or infiltration. The manipulation during surgical resection poses an additional acute irritational effect on the 1st and 2nd/3rd segments of the FN, which are at a high risk of intraoperative damage due to their particular vulnerability to stretching (lack of perineurium), and reduced resistance to compression (lack of epineurium) (Sekiya et al., 1985; Captier et al., 2005; Bendella et al., 2016). In the era of modern neurosurgery, newer “nerve-centered” surgical strategies have been developed achieving a higher FN preservation rate and functional outcome (Samii and Matthies, 1997b; Sampath et al., 2000). Intracranial FN injury results in facial palsy which has great impact on the psychosocial conditions of affected patients, and has a more severe clinical course than peripheral FN injuries (Wiet et al., 2001). When intraoperative damage occurs, direct coaptation of nerve ends or nerve substitution techniques are the currently preferred treatment, but are still associated with a poor functional recovery (Samii and Matthies, 1997b). The microsurgical techniques for FN palsies in the acute or secondary stage situation as well as additional experimental possibilities have drastically improved within the last decades. The effect of such interventions may differ in results and outcome when compared to intra- or extratemporal FN injuries (Captier et al., 2005; Burgette et al., 2012).

## Literature Data Collection

A systematic literature review was conducted in order to describe all available pertinent data. For the article selection process, the authors followed the recommendations made by the Preferred Reporting Items for Systematic Reviews and Meta-Analyses (PRISMA) (Moher et al., 2009).

### Search Strategy and Selection Criteria

Two authors (MR and ICH) independently performed a systematic literature review using PubMed, EMBASE and Medline as well as a manual review of the reference section of the provided article at two different time points. The last search was conducted before July 2020. The search strings in variable combinations were: “facial nerve regeneration AND surgery AND cerebellar; facial nerve injury AND skull base surgery; cerebello-pontine-angle AND cerebello pontine angle AND facial nerve recovery; cerebello pontine angle AND facial nerve recovery; cerebello pontine angle AND facial nerve; facial nerve AND outcome AND surgery AND vestibular schwannoma OR cerebellopontine angle meningioma; facial nerve AND animal model AND skull base; facial nerve regeneration AND animal model.” The reference lists of all identified sources were reviewed for additional relevant articles. The records were screened for duplicates and assessed for eligibility. All included reports contain information on FN state as well as follow-up evaluation of FN impairment. Only studies including data on FN palsy at least post-operatively were included. We did include studies with only partial information on FN function (i.e., only one post-operative assessment instead of the direct post-operative function, function on follow-up and therefore information on improvement). We excluded studies reporting on non-tumorous pathologies, single case reports, articles not reporting on FN function impairment or recovery at all, studies reporting on peripheral FN injury (beyond the fundus of the IAC), reviews and meta-analysis, studies only reporting recurring surgery, studies reporting a heterogenous cohort of treated pathologies, and cohorts which were presented in two studies. In cases where it could not be established with last certainty if specific patients were used in several studies from the same institution, we only included the publication with the largest cohort. After identifying reports on vestibular schwannoma (VS), representing the most frequent pathology with the highest number of cohort studies, we only included reports with information on extent-of-resection and the surgical approach. For the less frequent pathologies, this limitation was not possible, as many studies did not report on the extent-of-resection. We did not exclude studies in which patients went on to have further treatment modalities such as radiosurgery (especially gamma-knife) or radiotherapy.

### Outcome Measures and Data Extraction

**Figure 1:**
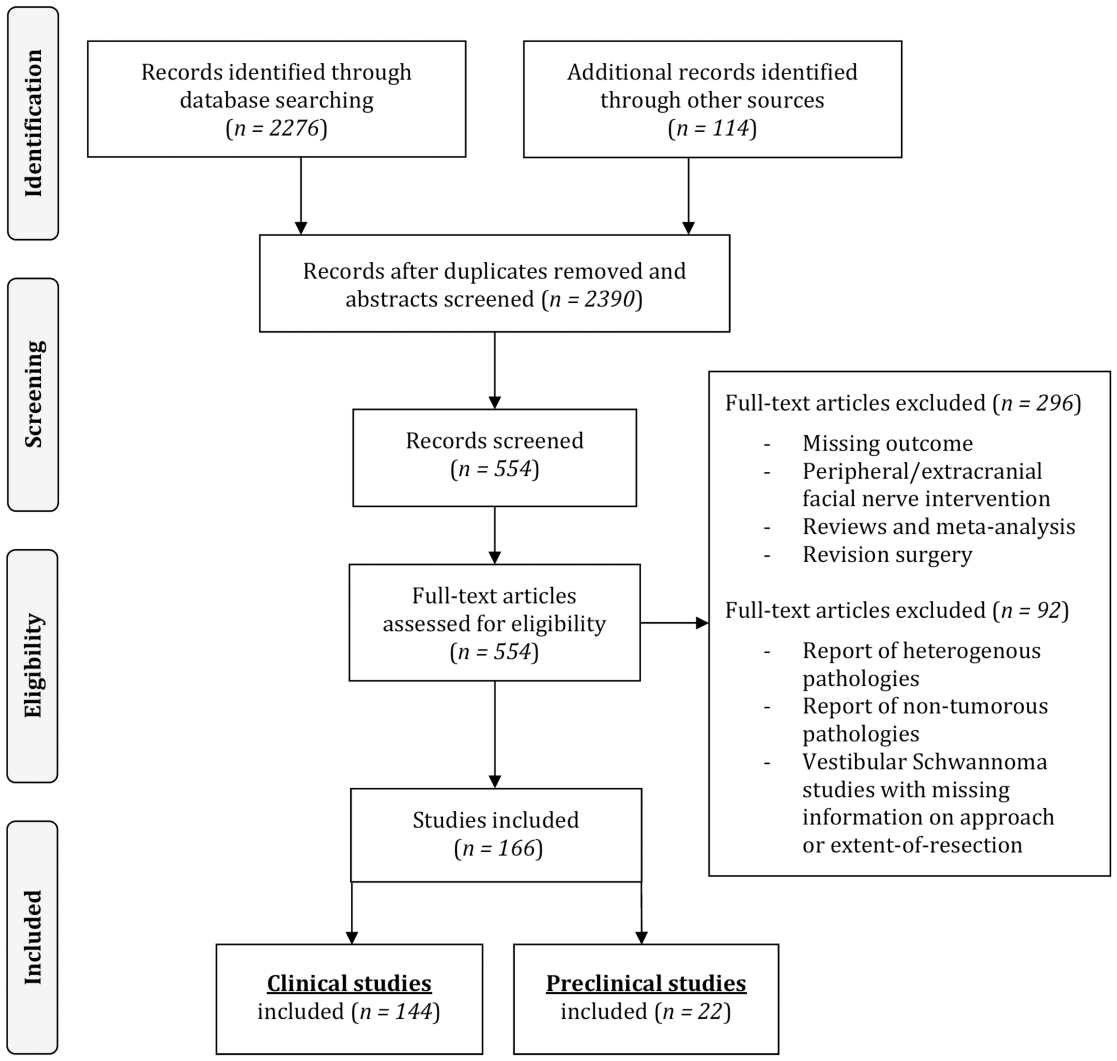
Article selection process.

The following information and variables were extracted from the included pre-clinical and clinical scientific articles: number of individuals, population from which the individuals are drawn, type of pathology, pre-operative FN status, surgical intervention (retrosigmoid [RSM], middle cranial fossa [MCF], translabyrinthine [TRL] approach and gross-total [GTR] or less-than-total resection), FN reconstruction technique in case of intraoperative injury, post-operative FN status and FN status on follow-up. FN function was assessed using the House and Brackman (HB) scale (House and Brackmann, 1985). A minority of studies combined HB I and II into good functional facial outcome. In these cases, this was considered as no FN impairment and full recovery. Included human clinical studies were reviewed and categorized according to the pathology, surgical approach and extent-of-resection, and pre-clinical studies according to the experimental animal. The process of selection is illustrated in Figure 1.

## Systematic Review of the Literature

Our systematic literature review identified a total of 2,388 papers. After full text review of the selected papers, a total of 166 publications published between 1961 and August 2020 were finally included. The resulting systematic overview on available clinical studies and pre-clinical animal models reports on intracranial FN preservation rates, causes of intracranial FN injury, FN reconstruction techniques and long-term recovery rates following CPA tumor surgery to elucidate gaps of knowledge, translational inputs, and discuss future research perspectives in this area.

### Clinical Studies on Facial Nerve Outcome After CPA-Tumor Surgery

Of the 166 included publications, 144 consisted of publications on 19,136 human subjects reporting FN impairment rate and outcomes after CPA tumor surgery (Figure 1).

#### Surgical Resection of Vestibular Schwannoma

Thirty-five studies report on FN outcomes after intentional complete resection of VS. Thirteen out of these studies (Nadol et al., 1987, 1992; Elsmore and Mendoza, 2002; Magnan et al., 2002; Mangham, 2004; Yang et al., 2008; Gerganov et al., 2009; Silva et al., 2012; Sharma et al., 2013; Setty et al., 2015; Roessler et al., 2016; Taddei et al., 2016; Hoshide et al., 2018) report on complete GTR-rates exclusively using the RSM approach in *n* = 944 individuals. About 41% of the individuals within this subgroup suffered from post-operative FN impairment, 31% displaying a moderate (HB grade III and IV) and 7% severe (HB grade V and VI) postoperative FN palsy. Thirty four percent of these patients improved on first follow-up to some degree and about 29% of those experienced complete recovery. In 6 studies (King and Morrison, 1980; Fisch et al., 1987; Moulin et al., 1995; Bento et al., 2002; Couloigner et al., 2003; Wang et al., 2013), a transpetrosal approach was solely used, whereas in the majority of studies (*n* = 264) a TRL approach was used. About 67% of these individuals suffered from post-operative FN impairment with 66% suffering from moderate FN palsy. Eighty percent of them improved on follow-up to some degree and again, out of those, 28% experienced a complete recovery of their FN function. Only two studies (Gantz et al., 1986; Raheja et al., 2016) report to have achieved complete GTR-rates exclusively via the MCF approach in *n* = 121 individuals, 34% of which suffered from post-operative FN impairment. Ten studies (Tator and Nedzelski, 1982; Kirkpatrick et al., 1993; Magliulo et al., 1998; Fenton et al., 2002; Colletti and Fiorino, 2003; Isaacson et al., 2005; Neff et al., 2005; Esquia-Medina et al., 2009; Matsui, 2012; Torres et al., 2017) report on complete GTR-rates in *n* = 734 individuals using a selected variation of surgical approaches. About 44% suffered from post-operative FN impairment, with 20% suffering from moderate and 13% from severe FN palsy in patients where information on HB grade was available. Forty four percent improved on follow-up to some degree. In 31% of patients who did have recovery of FN function, the recovery was complete (Table 1).

**Table 1:** Human clinical studies reporting facial nerve preservation and recovery rates after cerebellopontine angle surgery in gross-totally resected vestibular schwannoma using a singular approach or variable approaches: Quantitative synthesis of the population from which the individuals are drawn, interventions for facial nerve reconstruction, pre- and post-operative facial nerve status, and percentage of full recovery.

**Human studies**		**Intervention**			**Post-operative VII function**				**Improvement of VII function**	
**Vestibular schwannoma, gross-total resection**	**Patients**	**Surgical approach**	**GTR^#^ (%)**	**Nerve reconstruction technique for VII palsy after CPA-surgery**	**Post-operative VII impairment (%)**	**Good VII function (HB II) (%)**	**Moderate VII function (HB III and IV) (%)**	**Severe VII function (HB V and VI) (%)**	Recovery of VII function^*^ (%)	**Full functional recovery (%)**
**Retrosigmoid (*****n*** **= 13)**	*n* = 944									
Nadol et al. (1987)	69	RSM	100	None	35				88	63
Elsmore and Mendoza (2002)	127	RSM	100	33/44: XII/VII	74				17	17
Silva et al. (2012)	29	RSM	100	8 XII-VII	86	46	50	4		
Sharma et al. (2013)	72	RSM	100	None	56					
Gerganov et al. (2009)	99	RSM	100	2 EEA	44					
Roessler et al. (2016)	60	RSM	100	None	49	52	42	7		
Setty et al. (2015)	12	RSM	100	None	8	92			0	0
Taddei et al. (2016)	51	RSM	100	1 VA, 1 XII/VII	77	43	47	10		
Magnan et al. (2002)	119	RSM	100		8	97	4			
Mangham (2004)	73	RSM	100		3					
Nadol et al. (1992)	78	RSM	100		22				50	50
Yang et al. (2008)	110	RSM	100		25	84	10	6	25	18
Hoshide et al. (2018)	45	RSM	100		40				24	24
**Transpetrosal (*****n*** **= 6)**	*n* = 299									
Fisch et al. (1987)	6	TRL	100	3 CIG GANG, 2 CIG SN, fenestrated collagen splints	100		100		68	0
Wang et al. (2013)	25	TRL	100	CIG GANG, CIG SN, XII/VII	100				72	0
Couloigner et al. (2003)	35	TRL	100	None	57					
King and Morrison (1980)	150	TRL	100	XII/VII	67					
Moulin et al. (1995)	61	TRL	100	3 XII/VII	39	67	33			
Bento et al. (2002)	22	RL	100		27				100	83
**Middle cranial fossa (*****n*** **= 2)**	*n* = 121									
Gantz et al. (1986)	43	MCF	100	1 EEA	43					
Raheja et al. (2016)	78	MCF	100		24	90	10			
**Variable approaches (*****n*** **= 10)**	*n = 749*									
Fenton et al. (2002)	67	TRL, RSM, MCF	100		34				74	48
Neff et al. (2005)	74	TRL, RSM, MCF	100						11	11
Torres et al. (2017)	229	RSM, TRL, MCF	100		48	74	26		41	21
Magliulo et al. (1998)	60	RSM, TRL	100		25	72	22	7	17	3
Esquia-Medina et al. (2009)	96	RSM, TPTR, MCF	100		35	63	25	13	31	18
Colletti and Fiorino (2003)	50	RSM, MCF	100		62	71	25	4	55	55
Isaacson et al. (2005)	124	TRL, MCF	100		42	99	2		65	60
Kirkpatrick et al. (1993)	26	RSM, TRL	100		69					
Matsui (2012)	8	RSM, TPTR	100		13					
Tator and Nedzelski (1982)	15	TRL, MCF	100	EEA or XII/VII	64	71		29	56	

* *Recovery of function: Grade of improvement in HB after surgery or facial nerve reconstruction*,

# *percentage of patients that underwent gross-total-resection in the study-cohort*.

*CPA, cerebellopontine angle; GTR, Gross-Total-Resection; VII, facial nerve; RSM, retrosigmoid (trans-meatal); TRL, trans-labyrinthine; MCF, middle cranial fossa; TPTR, trans-petrosal; TRM, trans-mastoid; PS, presigmoid; ST, subtemporal; TO, trans-otic; TC, trans-cochlear; IFL, infra-labyrinthine; FL, far lateral; Tx, therapy; V/VII, trigeminal branch facial nerve anastomosis; XI/VII, spinal accessory-facial nerve anastomosis; VA, vestibular anastomosis; XII/VII, hypoglossal-facial nerve anastomosis or hemi-hypoglossal nerve transfer; EEA, end-to-end anastomosis; ESA, end-to-side anastomosis; SSA, side-to-side anastomosis; FNR, facial nerve rerouting; CIG, cable interposition graft; GANG, greater auricular nerve graft; SN, sural nerve; CFNG, cross facial nerve graft; GWI, gold weight implant; TRP, tarsoraphy; N/A, not applicable*.

Eighty-three studies report on VS surgery, where GTR was not achieved or an intentional less-than-total resection was accepted in some patients within the reported cohorts. Thirty out of these studies (Sugita and Kobayashi, 1982; Bentivoglio et al., 1988; Ebersold et al., 1992; Samii et al., 1992, 2006, 2010; Cerullo et al., 1993; Comey et al., 1995; Post et al., 1995; Colletti et al., 1997; Jung et al., 2000; Watanabe et al., 2003; Yamakami et al., 2004; Zhang et al., 2005; Sinha and Sharma, 2008; Misra et al., 2009; Gerganov et al., 2010; Zhao et al., 2010; Arlt et al., 2011; Haque et al., 2011; Nayak and Kumar, 2011; Pan et al., 2012; Iwai et al., 2015; Jeltema et al., 2015; Turel et al., 2015; Harati et al., 2017; Huang et al., 2017a,b; Zumofen et al., 2018; Taha et al., 2020) exclusively using the RSM approach report <100% GTR rates in *n* = 4,131 individuals. About 48.4% of the individuals suffered from post-operative FN impairment, of which 23.4% had moderate and 12.7% severe FN palsy. Fifty percent improved on follow-up to some degree with 30.1% of them making a full recovery (Table 2). In 14 studies (Pellet et al., 1987; Hardy et al., 1989; Charabi et al., 1994; Lanman et al., 1999; Mass et al., 1999; Sluyter et al., 2001; Mamikoglu et al., 2002; Brackmann et al., 2007; Godefroy et al., 2009; Piccirillo et al., 2009; Springborg et al., 2012; Moffat et al., 2013; Schwartz et al., 2013; Aristegui Ruiz et al., 2016), the TRL approach was used in *n* = 3,825 individuals. About 53.5% suffered from post-operative FN impairment, 22.9% suffered from moderate and 22% from severe FN palsy. 47.7% improved on follow-up to some degree and 16.9% of those experienced a complete recovery of the FN function. Three studies (Wigand et al., 1985; Haid and Wigand, 1992; Gjuric et al., 2001) report on the enlarged MCF approach in *n* = 1,061 individuals. About 36.1% suffered from post-operative FN impairment with 70% having moderate and 10% severe FN palsy (Table 3). Forty-one studies (Lye et al., 1982; Arriaga et al., 1993; Grey et al., 1996b; Moffat et al., 1996; Gormley et al., 1997; Sampath et al., 1997; Briggs et al., 2000; McElveen et al., 2000; Kaylie et al., 2001; Karpinos et al., 2002; Iwai et al., 2003; Mamikoglu et al., 2003; Roland et al., 2004; Anderson et al., 2005; Park et al., 2006; Seol et al., 2006; Bloch et al., 2011; Kazim et al., 2011; van de Langenberg et al., 2011; Martin et al., 2012; Nuseir et al., 2012; Raslan et al., 2012; Rinaldi et al., 2012; Yashar et al., 2012; Nonaka et al., 2013; Porter et al., 2013; Schmitt et al., 2013; Betka et al., 2014; Chen et al., 2014; Li et al., 2014; Tang et al., 2014; Kunimoto et al., 2015; Carlstrom et al., 2016; Golfinos et al., 2016; Monfared et al., 2016; Wise et al., 2016; Bernardeschi et al., 2018; Link et al., 2018; Mooney et al., 2018; Akinduro et al., 2019; Strickland et al., 2020) with *n* = 6,866 individuals used a selected variation of surgical approaches. About 90% of the individuals suffered from post-operative FN impairment. Of patients with FN impairment and available HB grade, 21.4% suffered from moderate and 15.7% from severe FN palsy. 51.5% improved on follow-up to some degree and 27.9% of them experienced a complete recovery of the FN function (Table 4).

**Table 2:** Human clinical studies reporting facial nerve preservation and recovery rates after cerebellopontine angle surgery with less-than total resection of vestibular schwannoma using the retrosigmoid approach: Quantitative synthesis of the population from which the individuals are drawn, interventions for facial nerve reconstruction, pre- and post-operative facial nerve status, and percentage of full recovery.

**Human studies**		**Intervention**			**Post-operative VII function**				**Improvement of VII function**	
**Vestibular schwannoma, single approach and less-than-total resection**	**Patients**	**Surgical approach**	**GTR^#^ (%)**	**Nerve reconstruction technique for VII palsy after CPA-surgery**	**Post-operative VII impairment (%)**	**Good VII function (HB II) (%)**	**Moderate VII function (HB III and IV) (%)**	**Severe VII function (HB V and VI) (%)**	Recovery of VII function^*^ (%)	**Full functional recovery (%)**
**RSM (*****n*** **= 30)**	*n* = 4,131									
Bentivoglio et al. (1988)	94	RSM	98.9		34	76	19	6		
Gerganov et al. (2010)	80	RSM	98.8	1 XII/VII	48	68	24	9		
Samii et al. (2010)	50	RSM	98.8	4/5 XII/VII	44	96	3	1		
Samii et al. (2006)	200	RSM	98.0	4 XII/VII, 1 SSA/SN	41	59	33	8	22	22
Ebersold et al. (1992)	255	RSM	97.6	7 EEA, 1 CIG, 7 XI/VII, 2 XII-VII, 2 conservative	48	64	23	13		
Samii et al. (1992)	61	RSM	96.7	5 XII/VII	31	69	16	16		
Sugita and Kobayashi (1982)	68	RSM	93.0	3 XII/VII	95	36	42	22	46	19
Post et al. (1995)	56	RSM	92.9		25	89	2	9	86	57
Comey et al. (1995)	83	RSM	89.0	17 XII/VII, 2 XI/VII, 4 conservative	84				52	
Zhang et al. (2005)	105	RSM	86.7	N/A	71	29	29	30	52	10
Huang et al. (2017b)	1167	RSM	86.2	79 EEA	84	36	63	1	64	12
Yamakami et al. (2004)	40	RSM	86.0	3 CIG, 1 XII/VII	16					
Nayak and Kumar (2011)	21	RSM	85,7	1 XII/VII	43	5	48	48		
Huang et al. (2017a)	657	RSM	84.6		67	33			43	43
Misra et al. (2009)	100	RSM	83.0		50	73				
Harati et al. (2017)	49	RSM	83.0		80	69	31	0	69	69
Turel et al. (2015)	100	RSM	78.0		86	99	1	0	35	13
Sinha and Sharma (2008)	58	RSM	75.9		8	43	40	17		
Jung et al. (2000)	30	RSM	73.3	2 GWI, 2 EEA	68	11	63	26		
Cerullo et al. (1993)	102	RSM	53.9		14	86	9	5		
Watanabe et al. (2003)	108	RSM	50.9		26	91	7	2		
Colletti et al. (1997)	88	RSM	45.5		58	72	20	9	29	2
Arlt et al. (2011)	50	RSM	44.0		40	57	21	23		
Zhao et al. (2010)	89	RSM	42.7		82	40	30	29	16	8
Haque et al. (2011)	151	RSM	36.0		4	97	3			
Taha et al. (2020)	95	RSM	27.4		33	67	16	17		
Jeltema et al. (2015)	55	RSM	18.0		71				41	41
Pan et al. (2012)	35	RSM	0.0		46					
Iwai et al. (2015)	40	RSM	0.0		23	95	5	1	89	67
Zumofen et al. (2018)	44	RSM	0.0		33	80	16	4	55	

* *Recovery of function: Grade of improvement in HB after surgery or facial nerve reconstruction*,

# *percentage of patients that underwent gross-total-resection in the study-cohort*.

*CPA, cerebellopontine angle; GTR, Gross-Total-Resection; VII, facial nerve; RSM, retrosigmoid (trans-meatal); TRL, trans-labyrinthine; MCF, middle cranial fossa; TPTR, trans-petrosal; TRM, trans-mastoid; PS, presigmoid; ST, subtemporal; TO, trans-otic; TC, trans-cochlear; IFL, infra-labyrinthine; FL, far lateral; Tx, therapy; V/VII, trigeminal branch facial nerve anastomosis; XI/VII, spinal accessory-facial nerve anastomosis; XII/VII, hypoglossal-facial nerve anastomosis or hemi-hypoglossal nerve transfer; EEA, end-to-end anastomosis; ESA, end-to-side anastomosis; SSA, side-to-side anastomosis; FNR, facial nerve rerouting; CIG, cable interposition graft; GANG, greater auricular nerve graft; SN, sural nerve; CFNG, cross facial nerve graft; GWI, gold weight implant; TRP, tarsoraphy; N/A, not applicable*.

**Table 3:** Human clinical studies reporting facial nerve preservation and recovery rates after cerebellopontine angle with less-than total resection of vestibular schwannoma using the trans-labyrinthine or the enlarged middle cranial fossa approach: Quantitative synthesis of the population from which the individuals are drawn, interventions for facial nerve reconstruction, pre- and post-operative facial nerve status, and percentage of full recovery.

**Human studies**		**Intervention**			**Post-operative VII Function**				**Improvement of VII function**	
**Vestibular schwannoma, single approach and less-than-total resection**	**Patients**	**Surgical approach**	**GTR^#^ (%)**	**Nerve reconstruction technique for VII palsy after CPA-surgery**	**Post-operative VII impairment (%)**	**Good VII function (HB II) (%)**	**Moderate VII function (HB III and IV) (%)**	**Severe VII function (HB V and VI) (%)**	Recovery of VII function^*^ (%)	**Full functional recovery (%)**
**TRL (*****n*** **= 14)**	*n* = 3,825									
Mass et al. (1999)	258	TRL	98.4		37	76	18	6		
Aristegui Ruiz et al. (2016)	417	TRL	98.6		30					
Hardy et al. (1989)	100	TRL	97.0	1 EEA, 6 XII/VII, 26 GWI, V/VII, CFNG	85	31	40	29		
Lanman et al. (1999)	190	TRL	96.3		61	55	22	23		8
Pellet et al. (1987)	224	TRL	96.0	None	13					
Mamikoglu et al. (2002)	81	TRL	95.1	4 EEA	81	25	15	60	59	15
Brackmann et al. (2007)	512	TRL	94.8	EEA, 3 CIG/GANG	49	71	15	4	38	17
Sluyter et al. (2001)	120	TRL	91.7	5 EEA, 6 CIG/SN, 8 secondary EEA	64	56	30	14		
Charabi et al. (1994)	23	TRL	91.3	XII/VII	65	35	4	61		
Springborg et al. (2012)	1,244	TRL	84.0		67	70	16	5	46	22
Schwartz et al. (2013)	400	TRL	81.3		54	57	12	31	48	23
Piccirillo et al. (2009)	57	TRL	82.5	7 CIG, 2 XII/VII	73	33	58	9		
Moffat et al. (2013)	148	TRL	66.0	14 EEA or CIG	47					
Godefroy et al. (2009)	51	TRL	26.0	None	22	78	22	0		
**MCF (*****n*** **= 3)**	*n* = 1,061									
Gjuric et al. (2001)	735	Enlarged MCF	97.1	3 CIG, 1 XII/VII, 1 EEA	72	92	7	1		
Haid and Wigand (1992)	263	Enlarged MCF	96.0	None	22					
Wigand et al. (1985)	63	Enlarged MCF	81.0	None	14					

* *Recovery of function: Grade of improvement in HB after surgery or facial nerve reconstruction*,

# *percentage of patients that underwent gross-total-resection in the study-cohort*.

*CPA, cerebellopontine angle; GTR, Gross-Total-Resection; VII, facial nerve; RSM, retrosigmoid (trans-meatal); TRL, trans-labyrinthine; MCF, middle cranial fossa; TPTR, trans-petrosal; TRM, trans-mastoid; PS, presigmoid; ST, subtemporal; TO, trans-otic; TC, trans-cochlear; IFL, infra-labyrinthine; FL, far lateral; Tx, therapy; V/VII, trigeminal branch facial nerve anastomosis; XI/VII, spinal accessory-facial nerve anastomosis; XII/VII, hypoglossal-facial nerve anastomosis or hemi-hypoglossal nerve transfer; EEA, end-to-end anastomosis; ESA, end-to-side anastomosis; SSA, side-to-side anastomosis; FNR, facial nerve rerouting; CIG, cable interposition graft; GANG, greater auricular nerve graft; SN, sural nerve; CFNG, cross facial nerve graft; GWI, gold weight implant; TRP, tarsoraphy; N/A, not applicable*.

**Table 4:** Human clinical studies reporting facial nerve preservation and recovery rates after cerebellopontine angle tumor surgery with less-than total resection of vestibular schwannoma variable approaches: Quantitative synthesis of the population from which the individuals are drawn, interventions for facial nerve reconstruction, pre- and post-operative facial nerve status, and percentage of full recovery.

**Human studies**		**Intervention**			**Post-operative VII function**				**Improvement of VII function**	
**Vestibular schwannoma, less-than-total resection and variable approaches**	**Patients**	**Surgical approach**	**GTR^#^(%)**	**Nerve reconstruction technique for VII palsy after CPA-surgery**	**Post-operative VII impairment (%)**	**Good VII function (HB II) (%)**	**Moderate VII function (HB III and IV) (%)**	**Severe VII function (HB V and VI) (%)**	Recovery of VII function^*^ (%)	**Full functional recovery (%)**
**Variable (*****n*** **= 41)**	*n* = 4,952									
Sampath et al. (1997)	611	RSM, TRL	99.8		48	62	20	18	42	42
Grey et al. (1996b)	276	RSM, TRL	99.6		64	54	28	18		
Gormley et al. (1997)	179	RSM, TRL, TPTR, TRM	99.4	1 EEA, 1 CIG, 1 refused Tx	26	77	22	1		
Arriaga et al. (1993)	515	TRL, MCF	98.6	None	40				41	
Betka et al. (2014)	333	RSM, TRL	98.5	12 CIG/GANG, 4 CIG/SN	58	55	30	15	41	17
Mamikoglu et al. (2003)	98	RSM, TRL	98.0		64	49	6	45	49	27
Kaylie et al. (2001)	93	RSM, TRL, combined, FL	97.8	XII/VII	28	81	15	4		
Briggs et al. (2000)	121	RSM, MCF	96.7		19	90	7	3		
Anderson et al. (2005)	67	RSM, TRL, or combined	95.5		77	45	22	34	49	21
Rinaldi et al. (2012)	97	RSM, TRL, RS, MCF	94.0		39	69	16	15	19	2
Lye et al. (1982)	33	RSM, TRL	93.9		67				50	50
Karpinos et al. (2002)	23	RSM, TRL, MCF	91.3		35	52	10	48		
Raslan et al. (2012)	47	RSM and TRL (staged)	87.7		55				46	46
Link et al. (2018)	143	RSM, TRL, MCF	85.3		78	83	16	1		
Moffat et al. (1996)	651	TRL, RSM	85.0	2 EEA, 1 CIG, 5 XII/VII, 2 CFNG	41	56	28	16		
McElveen et al. (2000)	100	RSM, TRL, MCF	81.0		13	93	4	4		
Roland et al. (2004)	54	RSM, TRL	75.9		36	84	10	6		
Schmitt et al. (2013)	267	RSM, TRL, MCF	74.9	None	59	59	20	21	57	30
Nonaka et al. (2013)	410	RSM, TRL, MCF	74.6	1 XII/VII, 3 EEA, 2 CIG/SN	14	86	11	3	58	
Tang et al. (2014)	262	TRL, RSM	74.1		40	78	14	7		
Golfinos et al. (2016)	202	TRL, RSM, MCF	73.8		27					
Nuseir et al. (2012)	232	TRL, RSM, MCF, TC	73.7		53	61	30	9		
Bloch et al. (2011)	624	TRL, RSM, MCF	72.9	None	42					
Carlstrom et al. (2016)	368	RSM, TRL, and MF	73.0	6 steroids, 35 steroids / antiviral	16				100	100
Martin et al. (2012)	216	RSM, TRL	68.1		54	25	24	51	21	21
Kunimoto et al. (2015)	9	RSM, TC	66.7	4 XII/VII	100	0	56	44	56	0
Porter et al. (2013)	153	TRL, RSM	52.0		39	75	22	3		
Wise et al. (2016)	37	RSM, TRL	49.0		27	97	3	0		
Kazim et al. (2011)	71	RSM, TRL	47.9	10 GWI, 4 XII/VII	38					
Yashar et al. (2012)	23	RSM, TRL	47.8		50	59	33	8		
Mooney et al. (2018)	9	RSM, TRL	22.2	1 GWI	56	22	22	56	67	22
Seol et al. (2006)	116	RSM, TRL	22.0	None	76	38	54	8	11	1
Park et al. (2006)	50	RSM, TRL	18.0		22					
Monfared et al. (2016)	73	RSM, TRL	16.0		33				35	14
Chen et al. (2014)	105	TRL, RSM, MCF	0.0		64	49	39	12		
Bernardeschi et al. (2018)	25	RSM, TRL	0.0		60	64	28	8	53	53
Iwai et al. (2003)	14	RSM, TPTR	0.0		29				100	0
van de Langenberg et al. (2011)	50	RSM, TRL	0.0		40	68	18	14	70	55
Li et al. (2014)	15	TM, MF	0.0		67	93	7	0	30	0
Strickland et al. (2020)	60	TRL, RSM, MCF	0.0	Wait and see	33	51	37	12	52	0
Akinduro et al. (2019)	34	RSM, TRL	0.0		7	85	12	3	86	71

* *Recovery of function: Grade of improvement in HB after surgery or facial nerve reconstruction*,

# *percentage of patients that underwent gross-total-resection in the study-cohort*.

*CPA, cerebellopontine angle; GTR, Gross-Total-Resection; VII, facial nerve; RSM, retrosigmoid (trans-meatal); TRL, trans-labyrinthine; MCF, middle cranial fossa; TPTR, trans-petrosal; TRM, trans-mastoid; PS, presigmoid; ST, subtemporal; TO, trans-otic; TC, trans-cochlear; IFL, infra-labyrinthine; FL, far lateral; Tx, therapy; V/VII, trigeminal branch facial nerve anastomosis; XI/VII, spinal accessory-facial nerve anastomosis; XII/VII, hypoglossal-facial nerve anastomosis or hemi-hypoglossal nerve transfer; EEA, end-to-end anastomosis; ESA, end-to-side anastomosis; SSA, side-to-side anastomosis; FNR, facial nerve rerouting; CIG, cable interposition graft; GANG, greater auricular nerve graft; SN, sural nerve; CFNG, cross facial nerve graft; GWI, gold weight implant; TRP, tarsoraphy; N/A, not applicable*.

**Figure 2:**
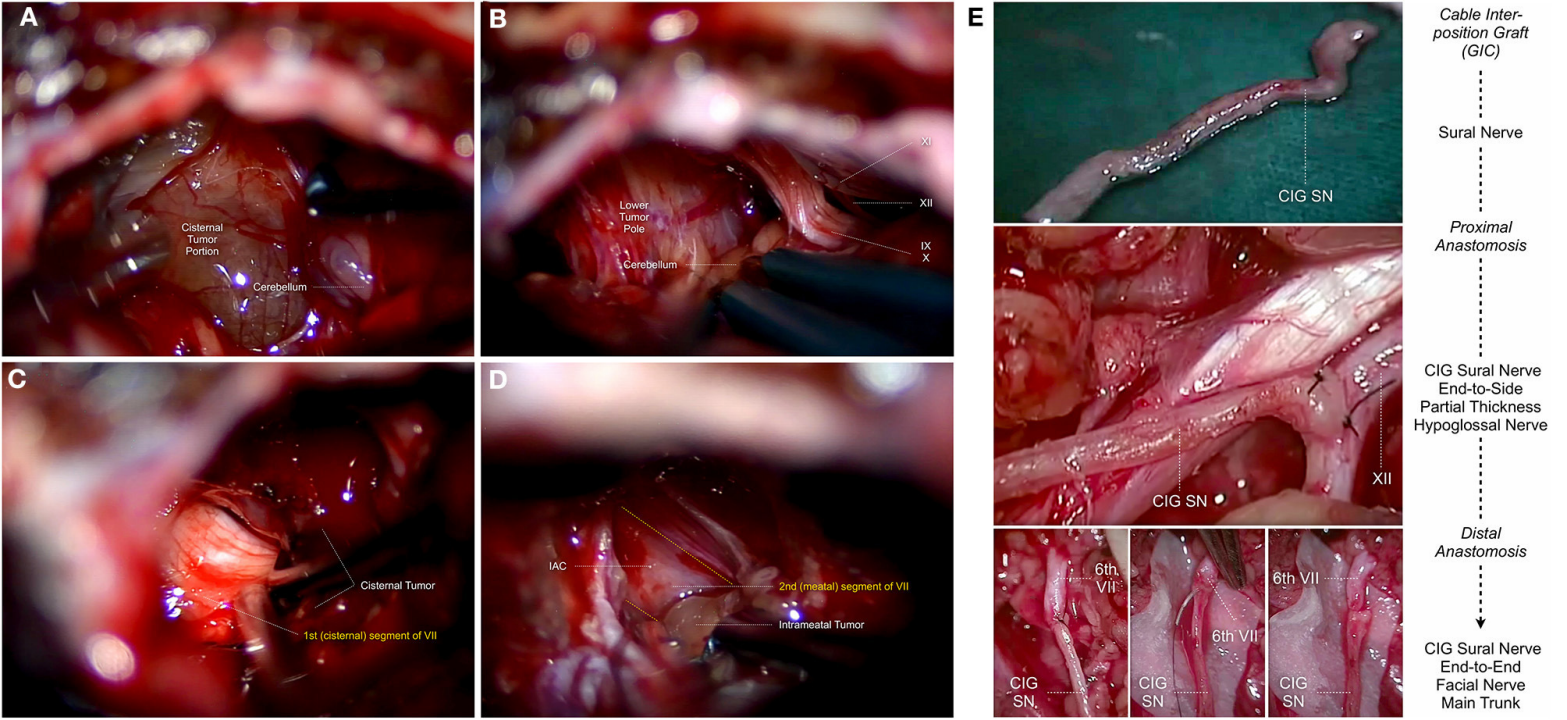
Topo-anatomical relationship of the intracranial facial nerve during human vestibular schwannoma surgery via the retrosigmoid approach: (**A)** Exposition of the posterior surface of the cisternal tumor portion after cerebellar retraction, and electrostimulation of the tumor capsule. **(B)** The lower pole of the cisternal tumor portion is in close relationship with the lower cranial nerves, consisting of the glossopharyngeal nerve [9th cranial nerve (IX)], the rootlets of the vagus nerve [10th cranial nerve (X)], the spinal accessory nerve [11th cranial nerve (XI)] and the hypoglossal nerve [12th cranial nerve (XII)]. The facial nerve (7th cranial nerve) is usually dislocated inferiorly toward the posterior aspect of the lower pole, where the proximal part of the nerve can be identified. **(C)** After sufficient internal tumor debulking, dissection of the tumor capsule away from the superomedial aspect of the 1st (cisternal) segment of the facial nerve [VII] is possible. The nerve is usually flattened and fanned by the tumor, increasingly adherent the more we move lateral and superior toward the internal acoustic porus. **(D)** After exposition of the 2nd (meatal) segment of the facial nerve using a high-speed diamond burr. Removal of the intra-meatal tumor portion from the 2nd (meatal) segment of the facial nerve [VII] up to the point, where the nerve exits the internal acoustic porus. The nerve is usually flattened but compact in this region and can be dissected from the tumor capsule. **(E)** Topo-anatomical relationship of the extracranial facial nerve during hypoglossal-facial nerve anastomosis using a sural nerve cable interposition graft. In case of intracranial interruption, protracted facial nerve palsy and anticipated tension of the anastomosis site, the use of an interposition sural nerve graft [CIG SN] between the distal (6th extratemporal) segment of the facial nerve [6th VII] and partial thickness of hypoglossal nerve [XII] is one of the most common facial nerve reconstruction techniques.

VS arise from the vestibular nerve and are essentially benign slow growing lesions, which continuously stretch the intra-arachnoidal FN as they grow. The intra-meatal portion of the FN is progressively compressed against the walls of the IAC (Bendella et al., 2016). Irrespective of their size, the tumor nerve interface is preserved by arachnoid layers, however, causing inflammatory processes and herniation of nerve fibers through the IAP (Bendella et al., 2016). As the tumor enlarges from the IAC, the FN is generally pushed anteriorly toward the middle cerebellar peduncle and the lateral pontine surface. In the remaining rare cases, it either courses through the tumor (3%), is infiltrated by tumor sheats (2%) or becomes enfolded by the tumor (1%) (Sampath et al., 2000). From its origin at the pons the nerve usually winds its way around the ventral superior aspect of the tumor in approximately 70% of cases, the ventral central aspect in 35% of cases, the ventral inferior aspect in 8% of the cases and in only <1 % will it be on the dorsal surface of the tumor (Sampath et al., 2000). The compression on the proximal intra-archnoidal FN segment is thought to compromise the blood supply of the more distal segment that, in addition, is stretched and fanned out. This results in a hypo- or atrophic intra-arachnoidal antero-lateral FN segment with a higher tendency to adhere to the tumor capsule (Carlson et al., 2012; Bendella et al., 2016). During surgery, the relaxation of the cerebellum is an important step, allowing excellent visualization of the entire posterior aspect of the tumor and both poles with minimal retraction. Great attention should be given to the maintenance of the arachnoid planes between the tumor, the cerebellum, the cerebellar peduncle and the brainstem, only using sharp dissection to cut the arachnoid layer. This layer should not be cauterized. This layer should not be cauterized. The posterior surface is stimulated in the rare instance that the FN has been displaced posteriorly (Figure 2A) (Sampath et al., 2000). The tumor is debulked extensively using an ultrasonic aspirator. The brainstem root-exit zone of the FN can be located at the lower pole, using low-intensity stimulation at 0.2 mA applied directly to the nerve (Figure 2B; Samii and Matthies, 1997a; Sampath et al., 2000; Samii et al., 2006). This step is critical for intraoperative verification of the integrity of the nerve along its entire length throughout the rest of the operation (Jeltema et al., 2015; Bernardeschi et al., 2018; Zumofen et al., 2018). In a next step, the attachment of the labyrinthine portion of the vestibular nerve between the tumor and the pons is cut to expose the FN that is further protected using patties as the tumor is debulked and the capsule is being continuously removed (Figure 2C). To identify the distal intra-meatal portion of the FN in the anterior superior quadrant, the IAC is drilled using a high-speed diamond burr and the IAP is exposed. After visual and electrophysiological identification with supramaximal stimulation, tumor dissection is generally possible without relevant adherence as the FN is flattened but remains compact (Figure 2D; Samii and Matthies, 1997a; Sampath et al., 2000; Samii et al., 2006; Jeltema et al., 2015; Bernardeschi et al., 2018; Zumofen et al., 2018). When exiting the IAP, the FN climbs the superior pole of the tumor and courses anteriorly. This anterolateral cisternal portion of the FN is highly vulnerable, as the nerve is invariably flattened and fanned out, tightly adhering to the tumor capsule in this protuberant portion (Samii and Matthies, 1997a; Sampath et al., 2000; Samii et al., 2006; Jeltema et al., 2015; Bendella et al., 2016; Bernardeschi et al., 2018; Zumofen et al., 2018). In most cases, the anteromedial tumor capsule can be dissected from the cisternal portion of the FN, which courses caudocranially along the medial tumor surface. This dissection is achieved under continuous stimulation with supramaximal output current. It is generally recommended to perform resection from a medial to lateral direction. Great care has to be maintained to ensure that the FN is not accidently injured as the upper pole is being debulked and dissected (Samii and Matthies, 1997a,b; Samii et al., 1997; Sampath et al., 1997, 2000). Frequently with larger tumor sizes, a remnant may be left along the surface leading anywhere from the brainstem to the IAP. Intended subtotal resection of VS, where intracapsular debulking of tumor was the main goal, was found in 8 out of 83 included studies (Iwai et al., 2003, 2015; van de Langenberg et al., 2011; Pan et al., 2012; Chen et al., 2014; Li et al., 2014; Akinduro et al., 2019; Strickland et al., 2020) reporting on *n* = 353 individuals (Tables 2, 4). Intended near-total resection, defined as maximal possible safe resection without endangering FN function, was found in 2 out of 83 included studies (Bernardeschi et al., 2018; Zumofen et al., 2018) with *n* = 69 individuals (Tables 2, 4). Special attention must be given to a predominantly cystic VS as the FN will often insinuate itself between folds of the tumor lobules, especially if the cyst has been decompressed. It is important in these cases to maintain the arachnoid plains meticulously and also refrain from releasing the tumor fluid prematurely (Charabi et al., 1994; Fundova et al., 2000; Jones et al., 2007; Eser Ocak et al., 2018). The arachnoid plain can often be detached to the point where the tumor interfaces with the cerebellum posteriorly and can gently be dissected and pushed into itself. Frequent stimulation of the anterior aspects of the tumor must be carried out to ensure the FN has not slipped between the clefts of the decompressed tumor (Charabi et al., 1994; Fundova et al., 2000; Jones et al., 2007; Eser Ocak et al., 2018).

#### Surgical Resection of CPA-Meningioma and Rare Pathologies

Thirteen of the included studies (*n* = 513 individuals) report on FN outcomes after surgery for CPA meningioma, 5 of which (Schaller et al., 1995; Roberti et al., 2001; Bassiouni et al., 2004; Roser et al., 2005; D'Amico et al., 2017) used the RSM approach exclusively in *n* = 196 individuals. About 27.1% suffered from post-operative FN impairment with 31.1% improving again on first follow-up to some degree and 58.3% experiencing a complete recovery. The remaining 8 studies (Sekhar and Jannetta, 1984; Thomas and King, 1996; Mallucci et al., 1999; Voss et al., 2000; Batra et al., 2002; Jiang et al., 2006; Baroncini et al., 2011; Nowak et al., 2013) report on a variety of selected approaches in *n* = 317 individuals. About 29.6% suffered from post-operative FN impairment with 54.8% improving on follow-up to some degree and 22% experiencing a complete recovery. In the overall cohort, FN palsy was rated as moderate in 13% and severe in 6%. CPA-Meningiomas maintain their respective plains, however, they display a tendency to engulf the nerves as they grow. Concerning CPA-meningioma it is important to be able to classify these tumors as being located either in the posterior, middle or anterior third of the petro-clival surface. The posterior only lesions are usually deemed resectable without further complications, as the cranial nerves are usually pushed anteriorly. The middle third lesions carry a higher risk for cranial nerve lesions, as they may be variable in their location, especially if the tumor is very large. Quite often however, the nerves are pushed and skirt the posterior pole of the tumor. The anteriorly located tumors are a challenge as the surgeon has to pass the instruments, especially the ultrasonic aspirator, beyond the nerves. Most of these meningiomas derive their blood supply from the tentorial dura and the petrous bone. Therefore, the first step is to coagulate and simultaneously debulk the superior aspects of the tumor. This corridor between the tentorium and the trigeminal nerve is gradually enlarged as the tumor is disconnected from its origin. This step is extended in a cranial caudal direction from the tentorium to the IAP and the FN is identified. The tumor is debulked superior to the FN. In tumors that extend anterior and inferior to the FN, the tumor parts between the FN and the lower cranial nerves are carefully debulked up to a rest of tumor anterior to the FN. Finally, the tumor attached to the deep aspects of the FN is dissected off the surface (Table 5; Sekhar and Jannetta, 1984; Schaller et al., 1995; Thomas and King, 1996; Mallucci et al., 1999; Voss et al., 2000; Roberti et al., 2001; Batra et al., 2002; Bassiouni et al., 2004; Roser et al., 2005; Jiang et al., 2006; Baroncini et al., 2011; Nowak et al., 2013; D'Amico et al., 2017).

**Table 5:** Human clinical studies reporting facial nerve preservation and recovery rates after cerebellopontine angle surgery for meningioma exclusively using the retrosigmoid approach or reporting variable approaches: Quantitative synthesis of the population from which the individuals are drawn, interventions for facial nerve reconstruction, pre- and post-operative facial nerve status, and percentage of full recovery.

**Human studies**		**Intervention**			**Post-operative VII function**				**Improvement of VII function**	
**CPA-meningioma, any resection grade (*n* = 13)**	**Patients**	**Surgical approach**	**GTR^#^^+^(%)**	**Nerve reconstruction technique for VII palsy after CPA-surgery**	**Post-operative VII impairment (%)**	**Good VII function (HB II) (%)**	**Moderate VII function (HB III and IV) (%)**	**Severe VII function (HB V and VI) (%)**	Recovery of VII function^*^ (%)	**Full functional recovery (%)**
**CPA-meningioma, any resection grade and single approach (*****n*** **= 5)**	*n* = 513									
Roberti et al. (2001)	9	RSM	100.0		22					
Roser et al. (2005)	72	RSM	86.1	3 XII/VII	28	75	8	17		
Bassiouni et al. (2004)	51	RSM	84.3	2 GWI	24				58	58
Schaller et al. (1995)	13	RSM	69.2		46	85	15	0		
D'Amico et al. (2017)	51	RSM	66.7		16				4	
**CPA-meningioma, any resection grade and variable approaches (*****n*** **= 8)**	*n* = 317									
Nowak et al. (2013)	48	RSM, TPTR	93.7		23					
Batra et al. (2002)	21	RSM, TRL	90.5		37	76	18	6	86	0
Voss et al. (2000)	40	TRL, TPTR, TC, MCF	82.0	2 XII/VII, 5 GWI, 6 TRP	30					
Jiang et al. (2006)	56	RSM, temporal-occipital/PS, temporo-occipital/ST trans-tentorial, temporo-occipital/supra- or infratentorial	78.6		32					
Thomas and King (1996)	41	RSM, TRL, TC, transtentorial	73.2		37				37	13
Mallucci et al. (1999)	20	RSM, TLR	65.0		25					20
Sekhar and Jannetta (1984)	22	RSM, ST	63.6		23				20	0
Baroncini et al. (2011)	69	RSM, TRL, MCF	44.9		30	90	9	1	77	77

* *Recovery of function: Grade of improvement in HB after surgery or facial nerve reconstruction*,

# *percentage of patients that underwent gross-total-resection in the study-cohort*.

*CPA, cerebellopontine angle; GTR, Gross-Total-Resection; VII, facial nerve; RSM, retrosigmoid (trans-meatal); TRL, trans-labyrinthine; MCF, middle cranial fossa; TPTR, trans-petrosal; TRM, trans-mastoid; PS, presigmoid; ST, subtemporal; TO, trans-otic; TC, trans-cochlear; IFL, infra-labyrinthine; FL, far lateral; Tx, therapy; V/VII, trigeminal branch facial nerve anastomosis; XI/VII, spinal accessory-facial nerve anastomosis; XII/VII, hypoglossal-facial nerve anastomosis or hemi-hypoglossal nerve transfer; EEA, end-to-end anastomosis; ESA, end-to-side anastomosis; SSA, side-to-side anastomosis; FNR, facial nerve rerouting; CIG, cable interposition graft; GANG, greater auricular nerve graft; SN, sural nerve; CFNG, cross facial nerve graft; GWI, gold weight implant; TRP, tarsoraphy; N/A, not applicable*.

Eight of the included studies (Fichten et al., 2006; Bakar, 2008; Sharma et al., 2008; Cho et al., 2009; Gunther et al., 2010; Mowry et al., 2012; Llorente et al., 2014; Ramos et al., 2015) report on FN outcomes after resection of non-vestibular nerve schwannomas in n = 364 individuals. Six of the included studies (Fisch et al., 1987; Borba et al., 2004; Makiese et al., 2012; Rotondo et al., 2014; Domenech Juan et al., 2016; Prasad et al., 2016) report on functional FN rates after the surgical resection of *n* = 101 temporal paraganglioma and *n* = 206 cholesteatomas (Table 6).

**Table 6:** Human clinical studies reporting facial nerve preservation and recovery rates after cerebellopontine angle surgery for non-vestibular schwannoma and rare pathologies using a heterogenous range of surgical approaches: Quantitative synthesis of the population from which the individuals are drawn, interventions for facial nerve reconstruction, pre- and post-operative facial nerve status, and percentage of full recovery.

**Human studies**			**Intervention**			**Post-operative VII function**				**Improvement of VII function**	
**Rare pathologies of the CPA**	**Patients**	**Treated Pathology**	**Surgical Approach**	**GTR^#^ (%)**	**Nerve reconstruction technique for VII palsy after CPA-surgery**	**Post-operative VII impairment (%)**	**Good VII function (HB II) (%)**	**Moderate VII function (HB III and IV) (%)**	**Severe VII function (HB V and VI) (%)**	Recovery of VII function^*^ (%)	**Full functional recovery (%)**
**Non-vestibular schwannoma, any resection grade, variable approaches (*****n*** **= 8)**	*n* = 364										
Sharma et al. (2008)	58	Trigeminal schwannoma	N/A	82.8		5.2				133	
Ramos et al. (2015)	15	VII schwannoma	N/A	100.0	CIG/GANG and fibrin glue	100.0	0	100	0	100	0
Fichten et al. (2006)	7	VII schwannoma	N/A	N/A	5 XII/VII	100.0				100	0
Mowry et al. (2012)	16	VII schwannoma	TRL, MCF	N/A	1 CIG	78.6	50	31	19	73	46
Gunther et al. (2010)	25	VII schwannoma	N/A	92.0	21 CIG/GANG or CIG/SN, 1 XI/VII	48.0	0	91	9	95	
Llorente et al. (2014)	34	Jugular fossa tumor	N/A	67.6	17 FNR, 17 conservative	55.9	29	39	33	91	18
Bakar (2008)	199	Jugular foramen schwannoma	N/A	79.9		13.1					
Cho et al. (2009)	10	Jugular foramen schwannoma	TPTR	80.0		60.0	40	30	20	83	
**Rare pathologies, any resection grade, variable approach (*****n*** **= 6)**	*n* = 313										
Fisch et al. (1987)	2	Glomus temporale tumor	Infratemporal	100.0	2 CIG/SN, fenestrated collagen splints	100.0		100		50	0
Borba et al. (2004)	24	Glomus jugulare tumors	IFL	95.8	One facial muscle transfer	4.2	88	13	0		
Makiese et al. (2012)	75	Glomus jugulare tumors	N/A	78.7	EEA	9.3				71	
Domenech Juan et al. (2016)	2	Epidermoid	MCF, TPTR, IT	100.0	None	0	100	0	0	100	100
Domenech Juan et al. (2016)	6	Cholesteatoma	MCF, TRM	N/A	None	33	66	34	0		
Prasad et al. (2016)	200	Cholesteatoma	TO, TC	100.0	53 FNR, EEA or CIG	47.8	53	26	23		
Domenech Juan et al. (2016)	1	Haemangioma	MCF	N/A	None	100	0	0	100		
Rotondo et al. (2014)	3	Cavernous malformation	N/A		None	33.0				100	100

* *Recovery of function: Grade of improvement in HB after surgery or facial nerve reconstruction*,

# *percentage of patients that underwent gross-total-resection in the study-cohort*.

*CPA, cerebello-pontine angle; GTR, Gross-Total-Resection; RSM, retrosigmoid (trans-meatal); TRL, trans-labyrinthine; MCF, middle cranial fossa; TPTR, trans-petrosal; TRM, trans-mastoid; PS, presigmoid; ST, subtemporal; TO, trans-otic; TC, trans-cochlear; IFL, infra-labyrinthine; FL, far lateral; Tx, therapy; V/VII, trigeminal branch facial nerve anastomosis; XI/VII, spinal accessory-facial nerve anastomosis; XII/VII, hypoglossal-facial nerve anastomosis or hemi-hypoglossal nerve transfer; EEA, end-to-end anastomosis; ESA, end-to-side anastomosis; SSA, side-to-side anastomosis; FNR, facial nerve rerouting; CIG, cable interposition graft; GANG, greater auricular nerve graft; SN, sural nerve; CFNG, cross facial nerve graft; GWI, gold weight implant; TRP, tarsoraphy; N/A, not applicable*.

### Causes of Intracranial Facial Nerve Injury After CPA-Tumor Surgery

Given the benign nature and slow growth potential of tumorous CPA lesions, the management strategy has to be tailored considering the biology and size of the lesion, age of the patient and the clinical situation. The most commonly reported pathology by far was VS, diagnosed in 17,946 (93.9%) individuals, followed by CPA-meningioma in 513 (2.6%) individuals and rare pathologies in 677 (3.5%) individuals, being consistent with previous reports (Grey et al., 1996a; D'Amico et al., 2017). Understanding the different growth patterns and their anatomical relationship to the FN is essential when surgical management is necessary (Sampath et al., 1997, 1998; Prasad et al., 2016; D'Amico et al., 2017). The current review revealed severe FN palsy in 7 to 15 % after vestibular schwannoma surgery, and 6% following the resection of CPA-meningioma (Tables 1–6). Preservation of FN function after CPA-tumor surgery ranges from 32 to 100% in the literature, and the functional outcome is inherently associated with the surgical approach, the size of the lesion, and the extent-of-resection that has been achieved during surgery (Charabi et al., 1994; Comey et al., 1995; Park et al., 2006; Seol et al., 2006). The most commonly reported surgical approaches were the RSM, the TRL and the MFC approach. In all of them, complete FN palsy occurred as a complication. Some of the included studies in the current review, however, present a FN preservation rate of 100% and do not report any complications (Mangham, 2004). Overall, some of these studies found the RSM approach to be more successful especially with regards to overall FN preservation, when compared to the MCF approach (Colletti and Fiorino, 2003; Mangham, 2004). Another possible approach which is commonly used in the included studies is the TRL approach. It has a similar complication profile as the RSM and the MCF approach (Lanman et al., 1999; Mass et al., 1999; Sluyter et al., 2001). VS size was found to be strongly associated with likelihood for post-operatively FN palsy with smaller tumors having a lower likelihood (Cerullo et al., 1993; Kazim et al., 2011).

The traditional paradigm of GTR of CPA tumors to minimize disease recurrence has to be balanced against the frequent occurrence of a permanent FN paresis/paralysis (King and Morrison, 1980; Gantz et al., 1986; Nadol et al., 1987, 1992; Bentivoglio et al., 1988; Moulin et al., 1995; Mass et al., 1999; Bento et al., 2002; Elsmore and Mendoza, 2002; Magnan et al., 2002; Couloigner et al., 2003; Mangham, 2004; Samii et al., 2006, 2010; Seol et al., 2006; Yang et al., 2008; Gerganov et al., 2009, 2010; Silva et al., 2012; Sharma et al., 2013; Wang et al., 2013; Setty et al., 2015; Aristegui Ruiz et al., 2016; Raheja et al., 2016; Roessler et al., 2016; Taddei et al., 2016; D'Amico et al., 2017; Hoshide et al., 2018; Tables 1, 5, 6). FN preservation surgery is justifiable, when acceptable extent-of-resection and good long-term control tumor rates are achievable (Seol et al., 2006; D'Amico et al., 2017; Bernardeschi et al., 2018; Zumofen et al., 2018). Segments of these tumors can be left *in-situ* if the surgeon judges the risk to critical structures unavoidable with further resections, provided there has been adequate decompression especially of the brainstem. In the modern era of neurosurgery, intraoperative neuromonitoring for functional integrity during resection, leads to a very high rate of macroscopically preserved intraoperative FN (Bendella et al., 2016). Furthermore, primary or adjuvant therapy options for tumor control, including radiation therapy, have gained crucial importance to achieve better functional outcomes. The concept of intended sub-total (Iwai et al., 2003, 2015; Seol et al., 2006; Bloch et al., 2011; van de Langenberg et al., 2011; Pan et al., 2012; Chen et al., 2014; Li et al., 2014; Akinduro et al., 2019; Strickland et al., 2020) and near-total tumor removal of vestibular schwannoma (Jeltema et al., 2015; Bernardeschi et al., 2018; Zumofen et al., 2018) and CPA-meningioma (D'Amico et al., 2017) hence favor functional outcome at a cost of further remnant disease management (Tables 2–5). The exact number of cases in the current review, in which neuromonitoring was used, remains unclear as this is not systematically reported. A previous study assessed the use of EMG facial monitoring with regards to FN injury and reported an improved preservation in cases where EMG facial monitoring was used (Taddei et al., 2016).

#### Facial Nerve Injury After CPA-Tumor Surgery

Although modern operative techniques allow macroscopic preservation of the FN during surgical resection in up to 98% of cases, the reported rates of post-operative FN palsies, presenting either immediately after the operation or with a delayed onset, range between 20–40% of cases (Arriaga et al., 1993; Batra et al., 2002; Carlson et al., 2012; Bendella et al., 2016). Injuries to the intracranial FN after skull base surgeries tend to be more severe than peripheral FN injuries (Mattsson et al., 1999; Wiet et al., 2001; Amine et al., 2014). Complete FN palsy rate (HB VI) was reported in up to 29% of cases, with 24% without recovery after 1 year in some studies (Wiet et al., 2001). The included clinical studies reported the direct type of lesion in only a limited number of publications. Consecutively, FN reconstruction was performed after evident FN palsy either directly during surgery, when electrical stimulation was impaired or lost, or when post-operative FN palsy was evident. The reporting of intraoperative visible FN injuries and lesions is generally underestimated and underreported in the literature. One reason for underreporting might be that the mechanism of FN injury is seldom clear unless the nerve is visibly damaged. This might explain the fact that in most cases of reported intraoperative FN injuries, sharp iatrogenic transection by neurotmesis was reported, suggesting that only a direct lesion was witnessed in these studies (Samii et al., 2006; Anaizi et al., 2014; Zumofen et al., 2018). Common non-reliable prognostic factors of FN injuries during skull base surgery of CPA tumors are tumor vascularity, the degree of FN flattening and the adherence along the tumor surface, as well as the preservation of the neural vascular supply (Lalwani et al., 1994; Bendella et al., 2016). Other putative causes described in the literature are viral reactivation, vasogenic edema or iatrogenic surgical damage. As previously discussed, the intra-arachnoidal FN has no true stabilizing support by the dura mater, and hence pinching, dissection and shifting of the nerve in various directions with single strong hits or repetitive low-impact compression most likely results in crush injuries by neurapraxia and axonotmesis (Bendella et al., 2016). In addition, severe stretching of any duration or longer stretching in general using spatula retraction toward the brainstem is thought to disrupt the stretched facial axons near the IAP, where the nerve has a strong stabilizing support by the dural folds. In pre-clinical experiments on rats, however, the intra-arachnoidal FN was shown to be very robust to stretching, which explains FN palsy to be rare as a presenting symptom, even when extreme dislocation and flattening of the nerve occurs by the tumor (Matthies and Samii, 1997; Bendella et al., 2016).

Transection of the FN by neurotmesis is most likely caused by the suction device or sharp instrument use. Ischemic or thermal lesions, resulting in coagulation necrosis, are thought to develop either during total petrosectomy and FN rerouting procedures, during dissection of the antero-lateral intra-arachnoidal FN from the tumor capsule, or with the use of an ultrasonic aspirator in the most anterior intra-tumoral aspects, where only marginal protection is left between the device tip and the hypo- or atrophic nerve (Sekhar and Jannetta, 1984; Tos et al., 1992; Schaller et al., 1995; Thomas and King, 1996; Mallucci et al., 1999; Voss et al., 2000; Roberti et al., 2001; Batra et al., 2002; Bassiouni et al., 2004; Roser et al., 2005; Jiang et al., 2006; Baroncini et al., 2011; Burgette et al., 2012; Nowak et al., 2013; D'Amico et al., 2017; Zumofen et al., 2018). If a direct lesion was not seen intraoperatively, the surgeon cannot be sure of the type of lesion that eventually occurred.

#### Intraoperative Facial Nerve Crush Lesion (Neurapraxia or Axonotmesis)

The mildest form of intraoperative FN injury during CPA tumor surgery is neurapraxia, where the continuity of the axons is preserved in the endoneurium causing a physiological conduction block and reversible changes (Sekiya et al., 1985; Bendella et al., 2016). Usually, nerve degeneration does not occur in this state. Neuroapraxia is caused by stretch deformation and compression, usually during dissection of the nerve from a tumor capsule (VS) or when the nerve is embedded within the tumor (meningioma). Stress strain studies have revealed an elastic limit of peripheral nerves up to 6–20% of their length. Under stronger force, axons in the endoneurial tubes might potentially disrupt and consecutively, nerve degeneration develops (Sunderland, 1981; Sekiya et al., 1985). A pre-clinical study by Bendella et al. (2016) compared stretch and crush injuries for the particular case of the intra-arachnoidal FN in rats. The authors concluded that crush injuries (with more than 50% of diameter compression) promote FN palsy, while brainstem displacement-induced stretching of the intra-arachnoidal FN trunk had no harmful effect on functional outcomes. The authors conclude, however, that human nerves might be more sensitive to stretch than the nerves of small rodents, thus limiting a generalized statement, and announce experiments in larger mammals.

Axonotmesis is a traumatic injury resulting in transection of the axons and the myelin sheath, while the fibrous protective structure (endoneurium) remains intact. The mechanical deformation of the neural membrane in these cases might be responsible for the conduction block secondary to this chronic state (Greiman and Lusk, 1991). A precise axonal reinnervation of the original peripheral targets after axonotmesis explains a generally good functional recovery in human patients and animal models (Hundeshagen et al., 2013). Experimental studies in rabbits on extratemporal FN crush injuries have shown loss of facial muscle activity followed by partial recovery after 2 weeks, and even complete recovery after 5 weeks from injury. However, the injured nerves displayed a lower partial cross-sectional axon density when compared to normal FN (Costa et al., 2006). Finally, it remains unclear whether cumulative low impact traumata, as they occur during large tumor resection surgeries, have the same impact as one single high impact event (Braun and Richter, 1996).

#### Intraoperative Ischemic Facial Nerve Lesion

The FN is vascularized by branches of the anterior inferior cerebellar artery, which provides the blood supply of the cochlear and labyrinthine artery within the IAC. After the geniculate ganglion, the superficial petrosal branch of the middle meningeal artery and the posterior auricular artery provide further blood supply. Rerouting of the distal FN segment during total petrosectomy, FN decompression and mobilization procedures, or for direct anastomosis after CPA-tumor surgery, have shown to compromise the blood supply at the geniculate ganglion and significantly impact functional outcome (Table 6; Llorente et al., 2014; Prasad et al., 2016). Acute compression produces ischemia of the FN as a consequence of endoneurial capillary occlusion. In case of large CPA-tumors, where the nerve is chronically compressed and stretched, collateral circulation might partially compensate ischemia as a relevant pathophysiological factor (Greiman and Lusk, 1991). Dissection of the FN from the tumor capsule, which is chronically compressed and stretched by a slow growing lesion, might disrupt the collateralized and neovascularized endoneurial capillary network and contribute to a sudden and unpredictable functional loss during surgery (Greiman and Lusk, 1991). This might be one explanation why no currently available technique allows neither for the reliable intraoperative assessment of the potential effect of the capsular dissection on the final post-operative FN function nor for the determination of a “point of no return,” beyond which the nerve will suffer permanent damage (Samii et al., 2006; Carlson et al., 2012; Anaizi et al., 2014; Zumofen et al., 2018). Experimental studies on ischemia-induced facial palsy models have identified ischemia as an important cause of facial synkinesis, and ephaptic transmissions between unmyelinated and myelinated axons as responsible for mass contracture during recovery (Takeda et al., 2008, 2015). Studies on hearing loss during CPA-tumor surgery have identified opening of the cisterns or suctioning of CSF to cause mechanical distortion of the anterior inferior cerebellar artery and the internal auditory artery at the junction, thus leading to silent cochlear ischemia (Tables 7, 8; Lusk et al., 1990). Even though sensory fibers are less resistant to pressure and ischemia than motor fibers, such phenomena might be relevant during the dissection of a chronically compressed and stretched FN during tumor surgery (Braun and Richter, 1996).

**Table 7:** Experimental studies exposing the facial (VII) or the vestibulo-cochlear nerve (VIII) complex in the cerebellopontine angle in small rodents (mice and rats): Quantitative synthesis of the population from which the individuals are drawn, surgical approach, type of intervention, and outcome.

**Small rodent animal models**			**Anesthesia**		**Surgical procedure**		**Type of experiment performed**		**Outcome**	
**References**	***n***	**Species**	**Initiation**	**Continuation**	**Surgical approach**	**CPA exposition**	**Intervention within the CPA**	**Outcome assessment**	**Surgical complication**	**Surgical outcome**
Bonne et al. (2016)	30	Mouse	Xylazine i.p. (10 mg/kg) Ketamine-HCl i.p. (100 mg/kg)		RSM	Stereotactic addressing of the VII/VIII complex only	Stereotactic injection of adult mouse Nf2^KO3/flox2^ Schwann cells (SC4-9luc)	Orthotopic grafting of cell lines allows study on pathogenesis of tumor-related hearing loss and pre-clinical drug evaluation	Transient ipsi- (surgical) and contralateral hearing loss due to opening of the arachnoidal layer at CPA to release CSF from cisterna pontis and/or magna	No death during or directly p.o., progressive weight-loss and euthanasia after 20 days (median survival 25 days)
Chen et al. (2019)	N/A	Mice	Ketamine i.p. (90 mg/kg) Xylazine i.p. (9 mg/kg) Buprenorphine s.c. (0.1 mg/kg)		RSM	Stereotactic addressing of the VII/VIII complex only	Stereotactical injection of mouse NF2^−/−^ -Schwann cells	Intravital imaging and hearing tests	i.o. bleeding during exposure. Thermal injury to cerebellum during drilling. Cerebellar damage due to excessive pressure. Bleeding from skull sinuses	Subsequent longitudinal imaging, neurological and hearing assessment for up to 2 months
Mattsson et al. (1999)	33	Rats	Chloral hydrate i.p. (35 mg/100 g)		TPTR MCF	Sufficient exposure of VII from the facial canal to the brainstem	Intracranial VII transection 0.5 mm from the brainstem	Degree of neuronal cell death (i.e., loss of motor neurons) and glial response in the facial motor nucleus	The operative procedure does not cause axonal damage	No signs of functional recovery of the injured VII during the observation period
Burgette et al. (2012)	14	Rats	Ketamine i.p. (100 mg/ml; 0.1 ml/100 g) Xylazine i.p. (20 mg/mL; 0.025 mL/100 g)		TPTR RSM	Sufficient circumferential exposure of VII/VIII complex	1 min intracranial VII crush injury using jeweler's forceps	Eye blink, flat vibrissae orientation, and absent vibrissae movement	Expected VIII loss due to intentional i.o. sacrifice	Complete p.o. facial nerve injury All animals recovered partial function (3 months). No complete recovery
Amine et al. (2014)	17	Rats								
Bendella et al. (2016)	30	Rats	Inhalational isoflurane (1.8%) O_2_ (0.6 l/min) N_2_O (1.2l/min)		RSM	Sufficient exposure of intra-arachnoidal VII	VII stretch injury by mechanical spatular brainstem displacement (1 or 3 mm) or electromagnet-controlled VII crush injury with a tweezers (closure velocity of 50 or 100 mm/s)	Whisking motor performance determined by video-based motion analysis.	Iatrogenic compression of the intra-arachnoidal VII by surgical/instrumental approach	Mechanical brainstem displacement without harmful effect. Light VII crush (50 mm/s) already resulted in paralyzed vibrissal muscles
Dinh et al. (2018)	10	Rat	Inhalational isoflurane (2–4%) O_2_ (1–2l/min)	Inhalational isoflurane (1–3%) O_2_ (1–2l/min)	RSM	Sufficient exposure of VII/VIII complex	Stereotactical injection of bioresorbable packing cut into 1 × 1 mm pieces (xenograft) to deliver mouse merlindeficient Schwann cells (SC4–9luc from Nf2 knockout mice, transformed to express luciferase)	Implanted mice develop tumors that involve the CPA/IAC imitating microenvironment for VS	Stereotactic and microsurgical techniques technically challenging and require years to master	2 rats developed severe head tilt, ataxia, and rapid weight loss. Life expectancy 21 days

*CPA, cerebello-pontine angle; O_2_, oxygen; NO, nitrous oxide; VII, seventh cranial nerve (facial nerve); VIII; eight cranial nerve (vestibulo-cochlear); VIIIc, eight cranial nerve (cochlear division); VIIIv, eight cranial nerve (vestibular division); IAC, internal acoustic canal; IAP, internal acoustic porus; IAA, internal auditory artery; AICA, anterior inferior cerebellar artery; EAEP, Early auditory evoked potentials; BEAP, brainstem evoked auditory potentials; CAP, compound action potentials; ARB, auditory brainstem response; AR, acoustic reflex; DPOAE, distortion-product otoacoustic emissions; p, power units; N, Newton; i.p., intraperitoneal; i.m., intramuscular; i.v., intravenous; i.o., intraoperative; p.o., postoperative; MRI, magnetic resonance imaging; SMF, stylomastoid foramen; CSF, cerebrospinal fluid; VS, vestibular schwannoma; Nf, neurofibromatosis*.

**Table 8:** Experimental studies exposing the facial (VII) or the vestibulo-cochlear nerve (VIII) complex in the cerebellopontine angle in large rodents (New Zealand rabbits and guinea pig): Quantitative synthesis of the population from which the individuals are drawn, surgical approach, type of intervention, and outcome.

**Large rodent animal models**			**Anesthesia**		**Surgical procedure**		**Type of experiment performed**		**Outcome**	
**References**	***n***	**Species**	**Initiation**	**Continuation**	**Surgical approach**	**CPA exposition**	**Intervention**	**Outcome assessment**	**Surgical complication**	**Surgical outcome**
Maurer and Mika (1983)	11	New Zealand rabbit	Xylacine	Phenobarbital i.v. and ether	RSM	Sufficient exposure of VII/VIII complex	1. Compression of the VIIIc with cotton 2. Interruption of the cochlear blood supply through IAA ligation	i.o. EAEP correlated with CPA mass volume and cochlear ischemia	Some damage of brain tissue from surgical procedure without EAEP changes	3 animals died during operation, 4 animals after 4–5 h (narcosis) and 1 after 3 days
Lumenta et al. (1988)	5	New Zealand rabbit	Atropine i.m. (0.16 mg) Xylacine i.m. (5 mg/kg) Ketamine i.m (25 mg/kg)	Ketamine i.v. 5 ml Xylacine i.v. 5 ml NaCl 0.9% i.v. 40 ml	RSM	Sufficient exposure of VII/VIII complex	Fogarty catheter placement (0.2, 0.4, or 0.6 ml) in the CPA	Reversibility of the BAEP changes correlated with CPA mass volume.	Retraction force of 54p or more caused necrosis at the lateral side of the cerebellum due to spatula pressure	0.4 mL: all survived 0.6 mL: bilateral BAEP loss and death
Lumenta et al. (1988)	10	New Zealand rabbit	Ketamine i.m. (50 mg/kg) Xylazine i.m. (10 mg/kg)	Supplemental doses of about 1/3rd of the original amount	RSM	Sufficient exposure of VII/VIII complex	Cerebellar retraction by a self-retaining retractor (7 mm) from a lateral to medial direction	Reversibility of the BAEP changes correlated with cerebellar retraction	N/A	Traction force (84 p): bilateral loss of waves III-V (BAEP) and death
Widick et al. (1994)	5	New Zealand rabbit					Compression of IAA at the IAP using directed glass micropipet with smooth bead end to block blood supply	DPOAE to estimate the cochlear blood flow changes correlated with compression of IAA		N/A
Braun and Richter (1996)	21	New Zealand rabbit	Ketamine i.v. (0.5 ml/kg of 6 ml with 150 mg) Xylazine (10 mg) Atropine s.c. (0.5 mg) Prilocaine s.c. (5 mg)	1.5 1/min oxygen with an inital dose of 2.5–3% halothane, reduced to 1.0–1.5%	RSM	Sufficient exposure of VII/VIII complex	CPA exposed to heated water with definitive increasing temperature (6 animals, 3 Min) IAA compressed with microdissector (6 animals, 3 Min) Constant pressure of l0 g (0.0.5N) on VIII (6 animals, 1 Min)	i.o. BAEP correlated with increasing temperature, IAA compression and constant pressure application on VIII	BAEP disappeared in 3 rabbits due to labyrinthal opening. BAEP did not react significantly below 71°C. Constant pressure of 10 g applied for 1 Min on VIII consistently caused loss of BAEP	Two rabbits died after induction of anesthesia
Telischi et al. (1999)	5	New Zealand rabbit	Ketamine i.m. (50 mg/kg) Xylazine i.m. (10 mg/kg)	Supplemental doses of about 1/3rd of the original amount	RSM	Sufficient exposure of VII/VIII complex	Compression of IAA at the IAP with micromanipulator directed glass micropipet with smooth bead end to block blood supply	Multiple 1 min compressions/interspersed 2-min rest periods to allow full recovery of the ABR or DPOAE	i.o. ABR latencies by microtraction on VIII during IAA compressions. VIII invariably traumatized at IAP by compression	N/A
Levine et al. (1993)	6	Guinea pig	Urethane (1,500 mg/kg) Innovar-Vet 0.25 (ml/kg)		RSM	Sufficient exposure of VII/VIII complex	Compression/transection of VII/VIII complex with 125-μm wire and/or cochlear nucleus with a blunt probe	Laser-Doppler measurements to estimate the cochlear blood flow changes	Immediate decrease in cochlear blood flow and velocity with VII/VIII compression	N/A

*CPA, cerebello-pontine angle; O_2_, oxygen; NO, nitrous oxide; VII, seventh cranial nerve (facial nerve); VIII; eight cranial nerve (vestibulo-cochlear); VIIIc, eight cranial nerve (cochlear division); VIIIv, eight cranial nerve (vestibular division); IAC, internal acoustic canal; IAP, internal acoustic porus; IAA, internal auditory artery; AICA, anterior inferior cerebellar artery; EAEP, Early auditory evoked potentials; BEAP, brainstem evoked auditory potentials; CAP, compound action potentials; ARB, auditory brainstem response; AR, acoustic reflex; DPOAE, distortion-product otoacoustic emissions; p, power units; N, Newton; i.p., intraperitoneal; i.m., intramuscular; i.v., intravenous; i.o., intraoperative, MRI, magnetic resonance imaging; SMF, stylomastoid foramen; CSF, cerebrospinal fluid; VS, vestibular schwannoma; Nf, neurofibromatosis*.

#### Intraoperative Facial Nerve Transection (Neurotmesis)

Within the described cases, transection injury by neurotmesis is less likely than other mechanisms of injury nowadays, and is mainly reported for surgical treatment of FN schwannoma and paraganglioma, where the FN was actively transected (Fisch et al., 1987; Amine et al., 2014). Ever since, attempts for a better functional outcome have led to FN sparing strategies, so that consecutively, this type of injury occurred less frequently (Samii and Matthies, 1997b; Sampath et al., 1997; Samii et al., 2006). However, suction was found to be a cause of FN transection during surgery and might still be a reason for such injury patterns nowadays (Tos et al., 1992; Burgette et al., 2012). Experimental studies with sharp intracranial FN injury revealed that FN continuity occurred with functional reinnervation of the target facial muscles, and electrophysiological findings were consistent with peripheral nerve regeneration (Glasby et al., 1995).

#### Intraoperative Thermic Facial Nerve Lesion (Coagulation Necrosis)

Clinical studies report thermic injury of the FN using high-speed drilling or the ultrasonic aspirator (Tos et al., 1992; Zumofen et al., 2018). Studies on hearing loss during CPA-tumor surgery revealed that thermal injury was only relevant, when the cochlear nerve itself or the feeding vessels suffer from coagulative necrosis (Braun and Richter, 1996).

#### Delayed Facial Nerve Impairment

A secondary deterioration of FN function after the immediately post-operative state as opposed to directly post-operatively can be observed in up to 29% (Sampath et al., 1998). The occurrence of delayed FN palsy is less common than immediate FN palsy (Grant et al., 2002). If late onset FN palsy occurs, improvement is very likely (Sampath et al., 1998). The exact reason for the delay is still poorly understood, however some studies suggest the advantages of steroid therapy in case of delayed FN palsy (Grant et al., 2002). One hypothesis about the pathophysiological mechanism is edema that only forms with a delay after the initial surgery and leads to a delayed compression of the FN subsiding further down the line and therefore leading to a high rate of improvement (Sampath et al., 1998). Other potential mechanisms include traction mediated by cerebellar swelling or CSF-leakage and immunological mechanisms.

### Intracranial Facial Nerve Recovery and Reconstruction Techniques After CPA-Tumor Surgery

Accounting for all included studies reporting on post-operative FN impairment in VS patients that underwent GTR, the rate of severe FN palsy (HB grade V and VI) ranges from 7 to 13%. The overall improvement rate was 53% (range 34–80%), with an overall complete recovery rate of 29% (range 28–31%) (Table 1). In studies, where less-than-total resection of a VS was accepted in some patients, severe FN palsy was reported in 15 % (range 10–22%) with improvement in 50% (range 48–52%) and overall complete recovery rate of 25% (range 16.9–27.9%) (Tables 2–4). Following resection of CPA-meningioma, an overall post-operative FN impairment of 28% was found, with severe FN palsy rates in 6%. The overall improvement rate was 43% (range 31–55%), with an overall complete recovery rate of 40% (range 22–58%) (Table 5). FN impairment most frequently presented after surgery. This is in accordance with the literature, where excision of VS has been associated with an interruption of the FN in around 2 to 10% of cases (Barrs et al., 1984; Fisch et al., 1987; Samii and Matthies, 1997a,b). In cases where a FN palsy does not recover, anastomosis or other forms of nerve repair are an option. Anastomosis can be performed immediately if transection is noted during surgery (Ebersold et al., 1992). It can also be done with a delay of several months in cases where complete FN palsy has not recovered, and electrophysiological examinations show signs of alteration. Treatment of FN disruption within the CPA has the goal of functional regeneration and restoration of facial symmetry. Pre-clinical data after peripheral FN injury in rats has shown, that the type of reconstruction significantly influences FN regeneration in terms of morphological and functional recovery. However, full functional recovery was not achieved, regardless of which reconstruction techniques was used (Guntinas-Lichius et al., 2007). The current review identified several strategies of FN reconstruction, including direct repair, cable nerve grafting, nerve substitution techniques and cross-face grafting. Especially if facial reanimation is done in a second separate step, more options emerge with many of them including bridging the gap using other nerves. Surgical techniques range from fascicular to epineural suture. However, due to its particular fascicular disorganization in the intracranial segment, and potentially reduced protective peri- and epineural layers, errors in fascicular orientation and connection result in a very high rate of dispersion of the distributed nerve fibers (Captier et al., 2005). One of the most important factors influencing and predicting recovery after intracranial FN injury is the timing of FN repair, with a significantly better outcome when the repair is performed earlier. Several potential methods have been reported over the last few decades, but only a few have found their way into daily clinical practice (Fisch et al., 1987). Irrespective of which method is applied to treat nerve transection, it should be chosen on an individual basis taking into account the surgeon's experience.

#### Direct End-to-End Facial Nerve Anastomosis

In case of interruption of the FN at the CPA or the IAC, the nerve continuity is often established by direct anastomosis. The current review detected several studies, where a direct end-to-end FN anastomosis was reported to be feasible after resection of VS using the RSM (Ebersold et al., 1992; Gerganov et al., 2009; Huang et al., 2017b), the TRL (Moffat et al., 1996, 2013; Sluyter et al., 2001; Mamikoglu et al., 2002; Brackmann et al., 2007), the MCF approach (Tator and Nedzelski, 1982; Gantz et al., 1986; Gjuric et al., 2001) (Tables 1–3) or variable approaches (Moffat et al., 1996; Gormley et al., 1997; Nonaka et al., 2013; Table 4), and following the resection of rare temporal bone lesions (Makiese et al., 2012; Prasad et al., 2016; Table 6). Experimental data suggests, that direct anastomosis of the FN leads to a higher rate of collateral branching and poly-innervation of the endplate when compared to other reconstruction techniques, resulting in decreased vibrissal movements in rats (Guntinas-Lichius et al., 2007). It has been shown that there is no statistical difference in results between micro-suture of the nerve ends or fibrin glue over the anastomosis at the CPA (Bacciu et al., 2009). The micro-suture is applied to the endoneurial ends of the nerves since the nerve at the CPA is devoid of epineurium (Captier et al., 2005). However, suturing at the CPA is cumbersome. Hence, the use of fibrin glue to oppose the nerve endings is more favored (Ramos et al., 2015). It should be noted that fibrin glue is applied over the anastomotic site of the two nerve endings and not in between the nerve gaps. It is also imperative to cover the anastomotic site with a fascia graft to prevent migration of the anastomosis into the nerve gap and consequently leading to a poor result. Reconstruction of FN at the CPA gives a HB grade III result at best in most cases (Samii and Matthies, 1997b). Change in facial tone is perceived after 3–5 months with early facial movements observed around 5–6 months after co-aptation. Recovery continues and is observed up to 12–15 months post-operatively.

#### Cable Nerve Grafting

In cases, where a segment of the FN is lost, a greater auricular or sural nerve graft is harvested and is used as an interposition graft. This strategy was identified in our review after VS resection using the RSM (Ebersold et al., 1992; Yamakami et al., 2004; Samii et al., 2006), TRL (Fisch et al., 1987; Sluyter et al., 2001; Brackmann et al., 2007; Piccirillo et al., 2009; Moffat et al., 2013; Wang et al., 2013), the MCF (Gjuric et al., 2001) approach (Tables 1–3) or using variable approaches (Moffat et al., 1996; Gormley et al., 1997; Nonaka et al., 2013; Betka et al., 2014; Table 4), after the resection of FN schwannoma (Gunther et al., 2010; Mowry et al., 2012; Ramos et al., 2015; Table 5), and following resection of rare temporal bone lesions (Fisch et al., 1987; Prasad et al., 2016; Table 6). Experimental data suggests a better functional and morphological outcome in rats when compared to direct FN anastomosis in terms of collateral branching and poly-innervation (Guntinas-Lichius et al., 2007). Distal stump rerouting has been advocated in order to gain 2–3 cm in length necessary for performing an end-to-end anastomosis (Brackmann et al., 1978; Arriaga and Brackmann, 1992). The current review identified FN rerouting only for the resection of temporal bone lesions including jugular fossa tumors (Llorente et al., 2014) and cholesteatoma (Prasad et al., 2016; Table 6). Although re-routing the distal segment of the FN to the CPA helps establishing nerve continuity through a single anastomosis as opposed to an interposition graft, it compromises the blood supply to the FN at the geniculate ganglion which negates the benefit of a single anastomotic site. Hence, in our view, it is preferable to use an interposition graft between the ends of the FN rather than reroute the distal FN to the CPA. Prasad et al. described a large series of 213 subjects who underwent interposition nerve grafting between proximal and distal FN stumps. Of these 50.7% subjects had a post-operative favorable HB Grade III outcome (Prasad et al., 2018). In the case of an auricular cable graft, the graft was harvested from a separate neck dissection and the sutured graft additionally secured with fibrin glue increasing the complexity and time for this approach (Wang et al., 2013; Ramos et al., 2015). The group reported a success rate of 100%, however, none of the patients achieved HB I, all patients recovered to either HB III or IV (Ramos et al., 2015). The duration of facial weakness was one of the most significant pre-operative factors affecting the final outcome (Prasad et al., 2018; Gao et al., 2019). The best outcomes were found if the interposition grafting is performed at the earliest but not later than 1 year after onset of facial palsy. Additional factors affecting the outcome include pre-operative grading of facial weakness, age and pathology. It was also found that intradural coaptation and extradural had more favorable outcomes compared to trans-dural coaptations (Prasad et al., 2018).

#### Hypoglossal to Facial Nerve Anastomosis

Hypoglossal to facial nerve (XII/VII) anastomosis was already described by Körte and Bernhardt (1903) at the beginning of the past century. Nowadays, XII/VII anastomosis is indicated when the proximal stump of FN at the brainstem is unavailable for grafting. In these cases, VII/XII anastomosis is usually planned within 2–3 months after the primary surgery for excision of vestibular schwannoma (Yawn et al., 2016; Gao et al., 2019). Early neurorrhaphy has been identified as a critical factor and is associated with better functional results than delayed neurorrhaphy (Yawn et al., 2016; Gao et al., 2019). VII/XII anastomosis is also used in cases of failed primary anastomosis where no facial movements are evident at 1 year post-reconstruction of the nerve at the CPA. This procedure should not be performed beyond 2 years after the onset of facial palsy since the motor end plates would by then be atrophied or fibrosed in most cases with a consequently poor result. The earliest case series of anastomosis between the FN and a full thickness hypoglossal nerve was described by Conley and Baker (1979). A complete transection of the hypoglossal nerve with and end-to-end anastomosis resulted in good functional results, however, with mass movements of the face in several studies (Pitty and Tator, 1992; Sood et al., 2000; Tanbouzi Husseini et al., 2013). This is supported by pre-clinical data, where significantly less collateral branching and poly-innervation of the endplates was detected in rats undergoing XII/VII anastomosis when compared to direct FN anastomosis (Guntinas-Lichius et al., 2007). However, the obvious disadvantage was a loss of hypoglossal function on the affected side with resultant paralysis of one half of the tongue. To obviate the disadvantage of complete tongue paralysis, many authors described anastomosis of the FN with split/partial thickness hypoglossal grafting (Lin et al., 2009; Samii et al., 2015; Dziedzic et al., 2018). By using such a reconstruction-technique, FN function improved, but there was still a morbidity associated with splitting or longitudinal sectioning of the hypoglossal nerve. Alternative to the end-to-end hypoglossal FN anastomosis, an end-to-side technique was described. Samii et al. (2015) compared the results of XII/VII end-to-end neurorrhaphy with XII/VII end-to-side neurorrhaphy. It was found that the VII/XII end-to-side neurorrhaphy with partial neurotomy of the hypoglossal nerve gives a functionally equivalent outcome when compared with full thickness VII/XII end-to-end anastomosis. However, the morbidity related to complete sectioning of the hypoglossal nerve is avoided (Samii et al., 2015). An experimental study by Liu et al. (2018) compared four different methods of XII/VII anastomosis in rats. The four methods included end-to-end neurorrhaphy, end-to-end neurorrhaphy using hemi-sectioned and longitudinally split hypoglossal donor nerve, end-to-side neurorrhaphy using a perineurial window and end-to-side neurorrhaphy with 30–40% partial neurotomy. They found that end-to-side neurorrhaphy with 30–40% partial neurotomy offers the best balance between the FN regeneration and donor deficits. The XII/VII-HFA technique has therefore gained widespread acceptance as the preferred method of VII/XII coaptation (Liu et al., 2018). In case of anticipated tension at the site of anastomosis, several studies report on the use of an interposition sural or greater auricular nerve graft between the distal segment of the facial and partial thickness of the hypoglossal nerve (Figure 2E; May et al., 1991; Flores, 2007; Volk et al., 2020). This combination of previously described techniques produced good facial movements without causing loss of tongue function. The resultant paresis of tongue movements on the affected side is easily compensated (May et al., 1991; Flores, 2007; Volk et al., 2020). There is no difference in facial outcome results between direct end to side anastomosis or interposition nerve graft. This was reported by Wang et al. (2013) by comparing sural grafting and XII/VII-anastomosis, where facial recovery was better in the sural graft compared to the XII/VII group in short as well as long term, although this difference did not reach statistical significance. Early facial movements are observed around 6 months post-operatively and recovery continues slowly up to one and half years post-operatively (Prasad et al., 2018). The current review detected a variety of studies reporting on XII/VII anastomosis after GTR of VS using the RSM (Silva et al., 2012; Chen et al., 2014; Taddei et al., 2016), the TRL (King and Morrison, 1980; Moulin et al., 1995; Wang et al., 2013) or the MCF (Tator and Nedzelski, 1982) approach (Table 1). XII/VII anastomosis is reported in less-than-totally resected VS cohorts using the RSM (Sugita and Kobayashi, 1982; Ebersold et al., 1992; Samii et al., 1992, 2006, 2010; Comey et al., 1995; Yamakami et al., 2004; Gerganov et al., 2010; Nayak and Kumar, 2011), the TRL (Hardy et al., 1989; Charabi et al., 1994; Piccirillo et al., 2009), the MCF (Gjuric et al., 2001) or using a variety of approaches (Moffat et al., 1996; Kaylie et al., 2001; Kazim et al., 2011; Nonaka et al., 2013; Kunimoto et al., 2015) as a common surgical strategy for FN reconstruction in the CPA (Tables 2–4). Two studies report the use of XII/VII anastomosis after resection of CPA-meningioma (Voss et al., 2000; Roser et al., 2005; Table 5), and Fichten et al. (2006) for the resection of facial schwannomas in 5/7 patients (Table 6).

#### Trigeminal to Facial Nerve Anastomosis

More recently, trigeminal to FN (V/VII) anastomosis has been advocated. Facial-masseteric anastomosis has gained popularity as a procedure that offers excellent facial movements without hemiglossal morbidity. The dynamic movements of the face are slightly superior in facial-masseteric anastomosis whereas facial-hypoglossal anastomosis offers a better result at rest (Altamami et al., 2019). The main advantages of using this co-aptation is the proximity of the masseteric nerve to the distal stump of the FN, its low morbidity, the early re-innervation and the ability to produce excellent mass movements of the face. Facial movements are observed as early as 4 months postoperatively and good facial function is usually achieved in about 12 months (Biglioli et al., 2017; Murphey et al., 2018). Alternatively, the middle deep temporal branches of the trigeminal nerve were recently proposed for facial reanimation, providing successful upper facial muscle reanimation with independent activation (Dauwe et al., 2016; Mahan et al., 2018). In the current review, we identified this technique after GTR of VS using the TRL approach by Hardy et al. (1989; Table 3).

#### Spinal Accessory-Facial Nerve Anastomosis

Spinal accessory-FN (XI/VII) anastomosis has been attempted to improve facial reinnervation. Indeed, the current review identified several studies using this reconstruction technique for FN reanimation after the RSM approach for VS (Ebersold et al., 1992; Comey et al., 1995; Table 2) and after resection of a facial neuroma (Gunther et al., 2010; Table 6). Ebersold and Quast (1992) report on a series of 25 patients who underwent anastomosis between the main trunk of spinal accessory and the FMN. It was found that 56% of subjects had good to excellent results and 80% of these subjects did not complain of relevant shoulder elevation problems. Poe et al. (1989) on the other hand described a modification to overcome the severity of shoulder elevation problems associated with XI/VII-anastomosis. In a cadaveric study, they identified the branch innervating the sternocleidomastoid muscle as an alternative nerve to perform such an anastomosis. However, XI/VII anastomosis never gained popularity, probably due to the paucity of fascicles available for robust reinnervation of the FN.

#### Cross Facial Nerve Grafting

One of the early descriptions of cross FN grafting was given by Scaramella (1975). However, it did not gain wide acceptance due to the limited facial movements that resulted from this technique. Harii et al. (1976) modified the technique by utilizing a free gracilis muscle transfer containing the obturator nerve, which is sutured to the sural nerve in a second stage after 6 months. The current review identified cross FN grafting as reconstruction technique after the RSM or the TRL approach for VS resection (Hardy et al., 1989; Moffat et al., 1996; Tables 3, 4). The cross FN grafting with free gracilis muscle transfer is the only option for creating a spontaneous dynamic smile for subjects with flaccid facial paralysis. It has demonstrated excellent results for improvement in oral commissure movement and is now considered one of the standard treatments (Bhama et al., 2014; Lindsay et al., 2014).

#### Gold and Platinum Weight Eye-Lid Implantation

One of the most troublesome sequelae of neglected paralytic lagophthalmos after FN palsy are corneal abrasions, keratitis, ulceration or corneal scarring with blindness in some cases (Jayashankar et al., 2008). To avoid these complications, care of the paralytic lagophthalmos is of great importance. Gold upper eyelid implants are one of the most popular methods used after facial paralysis, as documented in several of the included studies in the current review (Hardy et al., 1989; Jung et al., 2000; Voss et al., 2000; Bassiouni et al., 2004; Kazim et al., 2011; Mooney et al., 2018; Tables 2–4). In this procedure, the action of paralyzed orbicularis oculi is substituted by implanting a prefabricated gold implant in the upper eyelid that helps to shut the eye assisted by gravity when the levator palpebrae muscle relaxes. This procedure offers good cosmesis and excellent functional protection of the eye. It is also used as an adjunct to several nerve reinnervation procedures since eye closure due to the reinnervation takes time to develop. Once the eyelid function recovers post-reinnervation, the gold implant can be removed (Jayashankar et al., 2008; Siah et al., 2018). Subsequent to the success of gold implant, platinum implants were used for the same and are now extensively used. Platinum has a higher density than gold and is also considered more biocompatible. Several authors have described excellent results with platinum implants (Siah et al., 2018).

### Pre-clinical Experimental Studies on Intracranial Facial Nerve Injury

Of the included 166 publications, 22 studies were pre-clinical studies, in which an experimental surgical exposition of the CPA was feasible. Thirteen studies reported experiments in rodents consisting of mice or rats (Mattsson et al., 1999; Burgette et al., 2012; Amine et al., 2014; Bendella et al., 2016; Bonne et al., 2016; Dinh et al., 2018; Chen et al., 2019; Table 7), New Zealand rabbits (Maurer and Mika, 1983; Lumenta et al., 1988; Widick et al., 1994; Braun and Richter, 1996; Telischi et al., 1999) or guinea-pigs (Levine et al., 1993; Table 8). Nine studies (Chinn and Miller, 1975; Mangham and Miller, 1979; Sekiya et al., 1985, 1990; Fisch et al., 1987; Sekiya and Moller, 1988; Lusk et al., 1990; Greiman and Lusk, 1991; Glasby et al., 1995) reported experiments in larger non-rodent models (Table 9). Described surgical approaches where the RSM or the MCF approach. The vast majority of the studies involving cranial nerve manipulation in the CPA studied effects on hearing loss either by direct exposition and intervention, or creating tumors in the CPA in rodents (Maurer and Mika, 1983; Lumenta et al., 1988; Levine et al., 1993; Widick et al., 1994; Braun and Richter, 1996; Telischi et al., 1999; Bonne et al., 2016; Dinh et al., 2018; Chen et al., 2019) and non-rodent animal models (Chinn and Miller, 1975; Mangham and Miller, 1979; Sekiya et al., 1985, 1990; Sekiya and Moller, 1988; Lusk et al., 1990; Greiman and Lusk, 1991). Only a minority of studies did report on intracranial FN injuries in animal models (Fisch et al., 1987; Glasby et al., 1995; Mattsson et al., 1999; Burgette et al., 2012; Amine et al., 2014; Bendella et al., 2016). Mattsson et al. (1999) were the first ever to expose the intracranial FN in rats by a temporal craniotomy following the intratemporal FN by drilling the pars petrosa up to the IAP and successfully applying a very proximal axonotomy of the FN near the brainstem, resulting in a sharp intracranial FN injury. Burgette et al. (2012) and Amine et al. (2014) described a successful complete root exposure of the intracranial FN in the CPA via a RSM with creation of a 1-min crush injury mediated by a jeweler's forceps, creating a complete post-operative FN palsy in rats. More recently, Bendella et al. (2016) were able to apply a controlled mechanical brainstem displacement of 1 or 3 mm, resulting in a stretch injury by nearly doubling the intra-arachnoidal FN length from 2 to 3 mm up to 6 mm. In another subgroup of rats, an controlled crush injury of the intra-arachnoidal FN was applied using electromagnetically controlled watchmaker forceps mounted on a stereotaxic frame. Thus, the precise creation of a crush injury of 50% of the FN diameter was possible, with different compression velocities (Table 7).

**Table 9:** Experimental studies exposing the facial (VII) or the vestibulo-cochlear nerve (VIII) complex in the cerebellopontine angle in other animal than rodents: quantitative synthesis of the population from which the individuals are drawn, surgical approach, type of intervention, and outcome.

**Non-rodent model**			**Anesthesia**		**Surgical procedure**		**Type of experiment performed**		**Outcome**	
**References**	***n***	**Species**	**Initiation**	**Continuation**	**Surgical approach**	**CPA exposition**	**Intervention**	**Outcome assessment**	**Surgical complication**	**Surgical outcome**
Chinn and Miller (1975)	20	Cat	Phenobarbital sodium i.p. (35 mg/kg)	Phenobarbital i.v.	RSM	Sufficient exposure of VII/VIII complex	Fogarty catheter placement (5–7 mm) in CPA over VIII	i.o. BAEP changes correlated with VIII stretch by CPA mass	1. VIII injury during approach 2. Local ischemia due to balloon inflation	Minor mechanical trauma influence VIII function
Fisch et al. (1987)	12	Cat	Halothane, NO		MCF	Sufficient exposure of VII/VIII complex	Sectioning of VII and reanastomosis using collagen splints	Blink reflex	N/A	8/12 cats improved (blink reflex)
Lusk et al. (1990)	10	Cat	Ketamine i.m. (10 mg/kg) Atropine i.m. (0.04 mg/kg) Phenobarbital i.v. (25 mg/kg)	Supplemental phenobarbital (obliterated pedal / blink reflexes)	RSM	Sufficient exposure of VII/VIII complex	Stereotaxic manipulator to vertically align a weighted probe (1 g) with a 2-mm spherical mass to compress VII/VIII at the inferior rim of IAC	i.o. AR changes correlated with increased compression of VIII in the CPA	AR totally obliterated by masses of 7–9 g. VII in IAC was altered by masses of ≥10 g	N/A
Greiman and Lusk (1991)	12	Cat						i.o. ARB changes correlated with increased compression of VIII in the CPA	The amplitudes of all four ARB waveforms decreased with compression	Removal of skull and cerebellum resulted in increased ARB wave II amplitude
Sekiya et al. (1985)	12	Mongrel dog	Phenobarbital sodium i.v. (25 mg/kg)	Phenobarbital i.v. Tubocurarine chloride i.v. (0.1–0.2 mg/kg)	RSM	Sufficient exposure of VII/VIII complex	1. Cerebellar retraction with a tapered spatula (4 mm) latero-medial 2. Caudo-rostral retraction of VIII with a tapered spatula (2 mm) at the IAP	i.o. IAP-EAP/BAEP changes correlated with cerebellar/VIII traction	Mechanical vasospasm of the AICA-IAA complex	N/A
Sekiya et al. (1990)	26	Mongrel dogs	N/A	N/A	RSM	Sufficient exposure of VII/VIII complex	Cerebellar retraction with VII/VIII shifting caudal-to-rostral or rostral-to-caudal	Recording of i.o. AEP	N/A	Hemorrhages in area vestibularis of VIIIv, area cochlearis of VIIIc, preganglionic portion FN
Mangham and Miller (1979)	3	Macaque monkey	N/A	N/A	Extradural MCF	Sufficient exposure of VII/VIII complex	Inflatable balloon catheter placement in the CPA	Normative stapedius reflex, latency, amplitude, rise, decay, & relaxation correlated with CPA mass	One of the three had revision surgery and received a second implant	N/A
Sekiya and Moller (1988)	16	Rhesus monkey	Ketamine HC1 i.m. (8 mg/kg) Pentobarbital sodium i.m. (12.5 mg/kg)	Fentanyl citrate i.m. Droperidol i.m. (0.25 ml/kg)	RSM	Sufficient exposure of VII/VIII complex	Cerebellar retraction from a lateral to medial direction	i.o. BAEP/CAP changes correlated with cerebellar retraction	1. Hemorrhages at the fundus of the IAC (area cribrosa) through avulsion rupture of IAA branches 2. VIIIc avulsion at the basal turn of the cochlea (cribriform)	VIII fiber avulsion after cerebellar retraction/vertical mobilization. Organ of corti degeneration (vascular insufficiency)
Glasby et al. (1995)	6	Sheep	Diazepam i.v. (0.5 mg kg^−1^) Ketamine i.v. (2.5 mg kg^−1^)	Halothane (1.5–2.0% 1:2) with O_2_ and NO	RSM	Sufficient exposure of VII/VIII complex	Sharp division and 3 mm excision of VII rootles, repair with short freeze-thawed muscle autografts and secured in place with Tisseel fibrin glue	Blink reflex/snout symmetry. CNAP of the grafted CPA VII/buccal branch of VII, EMG of the buccinator muscle, cortical stimulation. Histology	No significant early or late complications apart from transient nystagmus and ataxia which resolved rapidly in every case.	100% facial nerve recovery rate, 83.3% full recovery after 1 year. Restoration of continuity, functional reinnervation of target muscles

*CPA, cerebello-pontine angle; O_2_, oxygen; NO, nitrous oxide; VII, seventh cranial nerve (facial nerve); VIII; eight cranial nerve (vestibulo-cochlear); VIIIc, eight cranial nerve (cochlear division); VIIIv, eight cranial nerve (vestibular division); IAC, internal acoustic canal; IAPIAP, internal acoustic porus; IAA, internal auditory artery; AICA, anterior inferior cerebellar artery; AEP, auditory evoked potentials; EAEP, Early auditory evoked potentials; IAP-EAP, evoked action potentials at the internal auditory porus of the cochlear nerve; BEAP, brainstem evoked auditory potentials; CAP, compound action potentials; ARB, auditory brainstem response; AR, acoustic reflex; DPOAE, distortion-product otoacoustic emissions; CNAP, compound nerve action potentials; EMG, electromyography; p, power units; N, Newton; i.p., intraperitoneal; i.m., intramuscular; i.v., intravenous; i.o., intraoperative; MRI, magnetic resonance imaging; SMF, stylomastoid foramen; CSF, cerebrospinal fluid; VS, vestibular schwannoma; Nf, neurofibromatosis*.

Fisch et al. (1987) used a MCF approach in cats to expose the cisternal and intra-meatal part of the FN and applied a sharp FN injury within the IAC. A sutureless anastomosis-technique using fenestrated collagen splints in one half of the cohort, and without using them in the other half, eventually led to a functional improvement of 75% of the operated animals in the longer term. Glasby et al. (1995) created a sharp injury of the intra-arachnoidal FN by sectioning 3 mm of the rootlets with a diamond knife and then used 5 mm freeze-thawed muscle autografts with coaxial orientation of the muscle and nerve fibers, fixing the construct suture-less by using fibrin glue. They reported a 100% functional recovery rate with 83.3% full recovery after 1 year in the operated animals (Table 9).

#### Anatomical Considerations for an Intracranial Facial Nerve Injury Model

Experimental studies using animal models play an important role in the evaluation of FN regeneration. The current review identified 22 experimental studies, where the surgical exposition of the CPA was described. Considering the limited space in the CPA and the challenge of any microsurgical manipulation of the cranial nerves, the size of the animal used to perform experiments is one limiting factor (Tables 7, 8). Larger species display similar nerve size and nerve regeneration rates as humans and thus allow for surgical interventions, which are very close to the clinical situation (Glasby et al., 1995). It has been shown that the microanatomy of the FN in larger mammals shares more similarities with humans than most of the used rodent models (Lu et al., 2015). This is especially true when assessing axonal regeneration, where larger animal sizes better recapitulate the distance and time (Table 9; Lu et al., 2015). There is a remarkable variation in vulnerability to axotomy, intensity of FN degeneration, neuronal cell death and regeneration processes between different species, and depending on animal age (Mattsson et al., 1999). Experimental studies, for example, found complete neuron recovery along with marked chromatolysis in rabbits a few weeks after FN crush injury, while mice developed atypical post-traumatic axon reactions (Costa et al., 2006). While the vast majority of experimental studies in the current review were found to deal with peripheral FN injuries distal to the FN trunk in its extratemporal portion, only six studies actually dealt with experimental intracranial FN lesion in an animal model (Tables 7–9; Fisch et al., 1987; Glasby et al., 1995; Mattsson et al., 1999; Burgette et al., 2012; Amine et al., 2014; Bendella et al., 2016). Further on, when taking brain size into account, CSF-production rates per body weight unit and CSF composition have been found to be very similar in rodents compared to humans (Spector et al., 2015a).

#### Small Rodent Animal Model

Based on the current review, mice were used for stereotactically guided injection of modified Schwann cells to create CPA-tumors (Bonne et al., 2016; Dinh et al., 2018; Chen et al., 2019). Bonne et al. (2016) accessed the petrous and occipital bone of mice using an anterior auricular flap and dissected the FN bluntly at its exit from the stylomastoid foramen. Bony removal over the parafloccular lobule was guided by the semi-circular canal and the lateral sinus. After dural opening, the parafluccular lobule was retracted postero-superiorly, and the transparency of the ampullae, situated close to the intra-arachnoidal FN, were used for stereotactic application of Schwann-cells into the CPA (Bonne et al., 2016). A similar concept in mice was recently published by Chen et al., where detachment of the cervical trapezius muscle, exposes the skull above the parafloccular lobule. The authors placed a 3 mm burr hole 2.2 mm lateral to the confluence of the sagittal and transverse sinuses, and 0.5 mm dorsal past the transverse sinus and were thus able to stereotactically lower a syringe 3.7 mm into the CPA for application of modified Schwann cells (Chen et al., 2019). Dinh et al. (2018) confirmed the same concept in rats, and were able to expose the root entry zone of the FN as well as the vestibulo-cochlear nerve at the brainstem. While these models are able to develop CPA-tumors to study the natural course of the disease and its influence on hearing function, any active manipulation of cranial nerves within the CPA seems to be hazardous. The feasibility of an intracranial and intratemporal FN manipulation in rats was published by Burgette et al. (2012), where a complete mobilization of the NF was necessary by approaching and decompressing the intratemporal FN from its exit from the stylomastoid foramen up to its tympanic segment. Following a RSM craniotomy and dural opening, the parafloccular lobule was retracted supero-medially, the craniotomy widened superiorly until the inferior cerebellar vein and mastoid cavity were identified, and then was widened posteriorly to create a 3 × 3 × 3 mm opening (Burgette et al., 2012). After identification of the petrous part of the temporal bone, the vestibulo-cochlear canal was opened and the vestibulo-cochlear nerve and the cochlea laterally were sacrificed in order to expose the posterior wall of the FN. Further decompression led to a circumferential view of the labyrinthine segment of the FN. Meticulous bony elevation of the remaining thin bone layer overlying the FN, anchored at the first genu, and hence susceptible to traction, was performed with a Rosen needle (Burgette et al., 2012). Finally, movements during application of the intracranial crush injury using a curved jeweler's forceps, were absorbed by the brainstem (Burgette et al., 2012). Amine et al. (2014) used the same technique in rats to test electrical stimulation and testosterone propionate on FN motoneuron survival following an intracranial FN crush injury. In the most recent of the included studies, Bendella et al. (2016) report successful exposition of the intra-arachnoidal FN in rats using a RSM approach by gentle retraction of the cerebellum and were able to apply mechanical displacement of the brainstem and stereotactical electromagnet-controlled crush injury close to the IAP (Table 7).

#### Large Rodent Animal Model

Based on the current review, the rabbit model seems to be the most frequently used model for manipulation of the FN in the CPA (Maurer and Mika, 1983; Lumenta et al., 1988; Widick et al., 1994; Braun and Richter, 1996; Telischi et al., 1999). The anatomical features of the rabbit FN have repeatedly been shown to be similar to those of the human FN (Costa et al., 2006). Maurer and Mika (1983) were the first ever to describe sufficient exposure of the FN and the vestibulo-cochlear nerve between the brainstem and IAP in New Zealand rabbits using a RSM. They were able to place cottonoids in the CPA, ligate the internal auditory artery and then measure the effect on hearing function. Lumenta et al. (1988) subsequently described the use of a self-retaining spatula to retract the parafloccular lobule medially and sufficiently expose the CPA, and were able to measure effects of brain retraction on hearing function. Further experiments confirmed the feasibility of the rabbit CPA-model by exposing the cranial nerves of the CPA and manipulating the internal auditory artery for hearing loss experiments (Widick et al., 1994; Braun and Richter, 1996; Telischi et al., 1999). Reported complications of the RSM were cerebellar necrosis due to spatula pressure (Maurer and Mika, 1983; Lumenta et al., 1988) as well as accidental opening of the labyrinth (Widick et al., 1994; Braun and Richter, 1996; Telischi et al., 1999). Levine et al. (1993) broadened the anatomical exposition with compression/transection of the FN and the vestibulo-cochlear nerve complex including the cochlear nucleus at the brainstem in a guinea pig model. However, aspiration of the cerebellum was necessary in these experiments (Table 8).

#### Other Animal Models

The earliest reports on animal models exposing the cranial nerves of the CPA by a RSM were by Chinn and Miller (1975) in a cat model. They were able to expose the CPA by removing the cerebellum and place a fogarty catheter imitating a mass lesion. As previously discussed, Fisch et al. (1987) described the MCF approach in cats with dissection of the middle fossa to reach the cisternal and intra-meatal part of the FN to perform a sharp intracranial nerve injury. Lusk and Greiman and Lusk et al. consecutively reported experiments in 22 cats, where they describe removal of the lateral cerebellum overlying the CPA with bipolar cautery and suction, with atraumatic dissection of the cisternal anatomy to expose the CPA, where they were able to directly compress the cranial nerves at the bony edge of the IAP (Lusk et al., 1990; Greiman and Lusk, 1991). A larger animal model was used by Sekiya and colleagues in several studies (Sekiya et al., 1985, 1990). The Mongrel dog was used to study the causes of cochlear, vestibular and FN injury during CPA-surgery and thus the authors were able to expose these nerves within the CPA in 39 dogs. Along with monitoring of the evoked action potentials of the internal auditory meatus portion of the cochlear nerve, auditory evoked brain stem responses and auditory evoked potentials, a histological analysis of the cranial nerves followed after medial retraction of the cerebellum and partial removal of the parafloccular portion, which was found to be in dorsal contact with the cranial nerves of the CPA. The authors were able to shift the CPA-nerve trunks in different directions (latero-to-medial, caudal-to-rostral or rostral-to-caudal direction) with cerebellar retraction using tapered spatulas. Caudal-to-rostral cerebellar retractions caused selective vestibular nerve damage in certain animals, with hemorrhage between the vestibular ganglion and the area vestibularis. Additionally, some animals displayed hemorrhage in the intrapetrosal preganglionic portion of the FN subjacent to the FN sheath (Sekiya et al., 1990). Rostral-to-caudal cerebellar retractions caused hemorrhage at the area cochlearis of the fundus of the IAC. The vestibular and FN, however, remained intact in these animals (Sekiya et al., 1990). The first ever described MCF approach was performed by Mangham et al. in Macaque monkeys, via extradural elevation of the temporal lobe. They identified a consistent anatomy similar to human specimens with the greater superior petrosal nerve as guidance and the basal turn of the cochlea, identified 0.5 mm anterior to the anterior limits of the IAC, as well as the labyrinthine portion of the FN, to reliably locate the IAP (Mangham and Miller, 1979). Sekiya and Moller (1988) consecutively described the RSM approach in Rhesus monkey, where they were able to expose the IAP after medial retraction of the cerebellum. They observed the parafloccular lobule to be in contact with the dorsal surface of the FN and vestibulo-cochlear nerves, potentially extending into the IAC. The lobule was thus removed to permit access to the CPA. Finally, Glasby et al. (1995) reported the largest animal model with an almost 1 year follow-up with experimental intracranial FN injury in sheep, where a medial cerebellar retraction was able to expose the IAP and the cranial nerves following an RSM approach. Microdissection of the FN covered by the superior vestibular nerve, was possible (Table 9).

#### Physiological Considerations for an Experimental Intracranial Facial Nerve Injury Model

The intra-arachnoidal FN has distinct microscopic differences with a more disorganized fascicular pattern and an arachnoid layer instead of the protective perineural and epineural connective tissue, when compared to the extratemporal FN divisions (Ishii and Takeuchi, 1993; Captier et al., 2005). Mattsson et al. (1999) found, that the loss of neuronal profiles following peripheral FN injury was generally higher after FN injury when compared to other peripheral nerves, suggesting a higher vulnerability of the FN to axotomy. They further confirmed a large discrepancy in neuronal degeneration with massive neuronal death (27% survival) as well as a marked presence of phagocytic microglia after intracranial FN axotomy, when compared to peripheral FN injuries (Mattsson et al., 1999). Further experimental studies have compared intra- and extratemporal FN crush injuries and found, that more proximal FN injuries result in a significantly higher loss of neuronal cell bodies in up to 15% with less cell survival within the relatively homogenous population of motoneurons in the facial motor nucleus of the pons (Marzo et al., 2010; Burgette et al., 2012). This finding was confirmed by Amine et al. (2014) with a 35% motoneuron loss after intracranial FN crush injuries (Table 7). Underlying pathophysiological explanations include a greater loss in axoplasmatic and axonal membranes with a short proximal stump, that may exceed the regeneration capacity of the injured neurons, and the loss of peripheral trophic support (Mattsson et al., 1999). A growing body of experimental evidence shows that efferent nerves whose cell bodies lie within the central nervous system can regenerate centrifugally under certain conditions. In particular, the environmental conditions at the regeneration site must sufficiently resemble the conditions around the distal portion of the nerve when it is still connected to the brainstem (Fisch et al., 1987; Glasby et al., 1995). Studies on FN crush injuries revealed that FN motoneurons ipsilateral to the injury displayed lower numbers of glutamatergic terminal and cholinergic perisomatic boutons along with atrophy and reactive microglial and astroglial cells in the facial nucleus of the brain stem, suggesting an ongoing degenerative process (Mattsson et al., 1999). However, poly-innervation of muscle fibers did not occur in certain studies, suggesting a precise target reinnervation (Hundeshagen et al., 2013). Such findings suggest that a restricted central nervous system plasticity and an insufficiency in afferent inputs to motoneurons are additional factors contributing to evident functional deficits after FN injury. These factors hence provide potential targets for treatment and augmentation by growth factors and influence of the neuronal microenvironment. Another relevant factor is the direct exposure of any artificial FN injury, consecutive reconstruction material, and biochemical regeneration processes to CSF in the subarachnoid space of the CPA, that follows a pressure gradient throughout its way to the dural sinuses (Spector et al., 2015a). As comprehensively reported by Spector et al. (2015b), there are major differences of the neuronal macro- and microenvironment between the subarachnoid CSF-filled space and the rest of the body. CSF has a stabilizing effect on homeostatic systems and regulates the extracellular environment of the central nervous system, which directly impacts the cells therein (Spector et al., 2015b). CSF has a distinct composition and contains inorganic ions, certain vitamins, glucose, and several organic molecules, as well as specific peptides and proteins (e.g. transthyretin, BDNF, IGF-2) (Spector et al., 2015b). When compared to plasma, for example, CSF is slightly more acidic (pH 7.33 vs. pH 7.4), and has a relative lack in white cells and immunoglobulins (Spector et al., 2015b). Along with the cisternal anatomy of the CPA, CSF has very distinct supportive (mechanical) and protective functions. The high turn-over and circulation rate stabilizes the biochemical and hemodynamic neuronal microenvironment and efficiently removes metabolic end products from and transports neuroendocrine factors within the central nervous system. The pulsatile and high turnover of CSF itself acts as part of the immunological protection (Spector et al., 2015b). This particular physiological macroenvironment has to be considered when taking intracranial FN injury models into account, as the CSF therefore is able to remove neurotrophic factors and other mediators of inflammation and regeneration. The intracranial FN is myelinated by Schwann-cells very close to the root exit zone at the brainstem, the so called Obersteiner-Redlich zone, and is therefore considered as part of the peripheral nerve system (Guclu et al., 2009). Biochemical processes of regeneration and guidance at the free floating nerve stumps and axonal endings, that usually do not have contact to CSF, might hence experience a different microenvironment, when compared to peripheral FN injuries (Sawamoto et al., 2006; Spector et al., 2015b). Additionally, the microsurgical opening of the arachnoid cisterns, blood products, scar formation, cavitation and inflammatory processes from the surgical/experimental process itself have to be considered. They most likely account to worsening of clinical or experimental conditions and impairment by increasing neuronal loss and glial reaction, which eventually impact reliable results in clinical and experimental studies (Spector et al., 2015a; Biscola et al., 2017).

## Anatomical Feasibility to Expose the Intracranial Facial Nerve in a Rabbit Animal Model

Based on the current review, the rat (Bendella et al., 2016) and the rabbit model (Maurer and Mika, 1983; Lumenta et al., 1988; Widick et al., 1994; Braun and Richter, 1996; Telischi et al., 1999) seem to be the most feasible rodent models for manipulation of the intra-arachnoidal FN in the CPA. Mice were used for stereotactically guided injection of modified Schwann cells to create CPA-tumors (Bonne et al., 2016; Dinh et al., 2018; Chen et al., 2019). Anatomical information on the rabbit CPA model in these mainly historical articles remains scarce (Table 8; Maurer and Mika, 1983; Lumenta et al., 1988; Widick et al., 1994; Braun and Richter, 1996; Telischi et al., 1999), especially in terms of visualization and detailed anatomy. In a more recent cadaveric study by our group, seven pigmented young adult female rabbits (New Zealand genus), that have undergone euthanasia within a study protocol not involving the central nervous system, served as post-mortem subjects to expose the intra-arachnoidal FN and recapitulating the available information from the identified rabbit models (Table 7). To obtain detailed anatomical information of the IAP, IAC, and FN canal of the petrous bone, the animals were perfused with warm normal saline to expel blood via a thoracotomy and cannulation of the left cardiac ventricle, and perfusion-fixed with formalin-calcium acetate (Roethlisberger et al., 2015).

### Radiological Assessment

**Figure 3:**
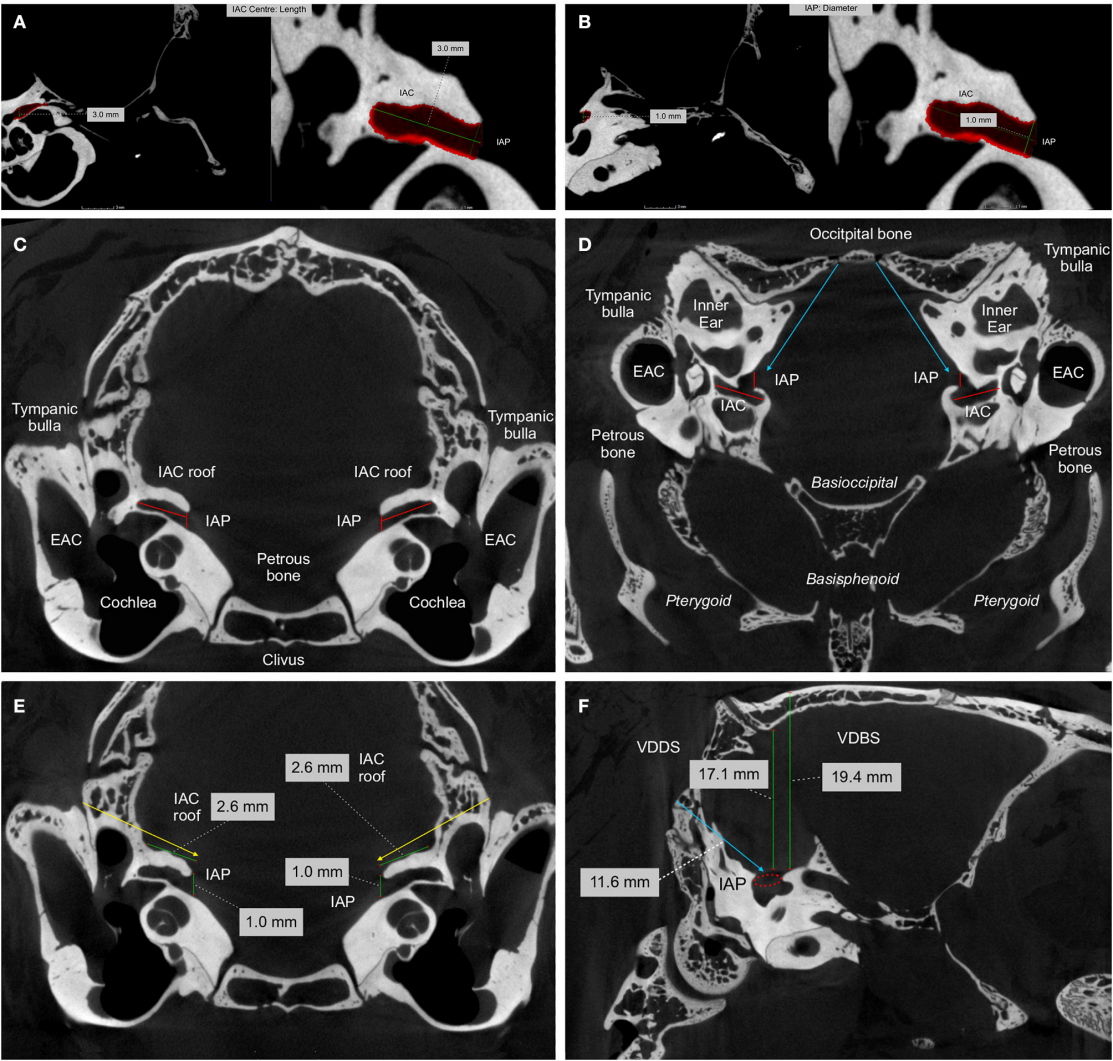
High resolution computed tomography (HRCT) imaging of a perfusion fixed New Zealand rabbit skull: The images were generated using the nanotom^®^m system rendering images. Spatial measurements of the length of the internal acoustic canal [IAC] and the internal acoustic porus [IAP] are based on all three cross-sections. The middle cranial fossa approach (yellow arrows) and the retrosigmoid trans-meatal approach (blue arrows) are indicated. **(A,B)** Spatial measurements together with a three-dimensional rendering of the manually segmented anatomical structure shown in red. Assessment of length along the central IAC and the diameter of the IAP, defined as the most medial border of the IAC toward the intracranial compartment. **(C)** Coronal; **(D)** Axial HRCT cross-sections defining the IAC, the IAP [red lines] and the bony roof of the IAC. **(E)** Coronal HRCT cross-section used for spatial measurement of the IAC, the IAP and the bony roof of the IAC [green lines]. **(F)** Sagittal HRCT cross-section used for the spatial measurement of the vertical distance from the IAP to the interparietal bony [VDBS] and dural surface [VDDS] and the distance of the IAP to the occipital (retromastoid or retrosigmoid) bony surface [blue line] (Roethlisberger et al., 2015).

In our earlier cadaveric study, rabbit skulls were used for radiological high-resolution assessment (Roethlisberger et al., 2015). The laboratory-based absorption-contrast micro computed tomography was carried out on the nanotom^®^m (GE Sensing & Inspection Technologies GmbH, Wunstorf, Germany) system, which is equipped with a 180 kV nanofocus transmission source and a 3,072 × 2,400 pixels GE DXR detector. For all the performed measurements, the tube acceleration voltage was set to 150 kV, the beam current was set to 50 μA, the effective pixel size was 40 μm and the total scanning time was 180 min. To reduce the effect of faulty detector pixels on the projections, the acquired radiographs were filtered with a 2D median filter with a 3 × 3 kernel prior to reconstruction. The filtered radiographs were subsequently reconstructed using the nanotom^®^m manufacturer's software phoenix datosx 2.0.1–RTM (GE Sensing & Inspection Technologies GmbH, Wunstorf, Germany) by means of a modified Feldkamp cone beam reconstruction algorithm. Subsequently, the datasets were imported in the VGStudio MAX 2.1 (Volume Graphics GmbH, Heidelberg, Germany) software for the assessment and measurement of the anatomical structures of interest based on simple thresholding and manual segmentation (Figures 3A,B), and three-dimensional rendering of the acquired tomograms. The high-definition CT-scans (nanotom^®^ m) of adult New Zealand rabbits revealed an average IAP diameter of 1.0 mm (range 0.8–1.2 mm) and a cross-sectional area of 1.1 mm (range 0.3–2.2 mm). The average IAC length was 2.9 mm (range 2.5–3.5 mm). The bony IAC roof had an average of 2.6 mm in length (range 1.4–3.4 mm) with an average thickness of 1.5 mm (0.9–2.3 mm). The distance from the interparietal bone surface to the IAP, relevant when discussing the MCF approach concept, was 19.4 mm (range 18.1–21.8 mm). The distance from the retrosigmoid bony surface to the IAP, relevant when discussing the RSM approach concept, was 11.6 mm (range 10.9–12.5 mm) (Figures 3C–F; Table 10).

**Table 10:** High resolution computed tomography (HRCT) imaging using the nanotom^®^m system: bilateral spatial measurements of selected anatomical regions in seven individual (I-VII) perfusion fixed New Zealand rabbit skull bases.

**Anatomical region**	**Measurements**	**Side**	**I**	**II**	**III**	**IV**	**V**	**VI**	**VII**	**Average**	**Average L/R**
**Internal acoustic porus**	Diameter (mm)	L	0.9	1.0	1.1	1.1	1.0	1.0	1.0	1.0	
		R	1.0	1.0	1.2	1.1	0.9	1.1	0.8	1.0	1.0
	CSA (mm^3^)	L	0.8	1.2	1.4	0.6	0.7	1.0	1.2	1.0	
		R	2.2	1.2	1.6	0.3	0.7	0.7	1.2	1.1	1.1
	**Middle cranial fossa approach**										
	VDDS (mm)	L	17.9	17.1	19.0	15.8	16.9	16.9	16.7	17.2	
		R	17.7	17.3	19.1	15.7	16.7	16.5	16.2	17.0	17.1
	VDBS (mm)	L	19.5	20.2	21.8	18.5	19.2	18.5	18.5	19.5	
		R	19.5	20.4	21.5	18.5	19.0	18.1	18.1	19.3	19.4
	**Retrosigmoid trans-meatal approach**										
	Retromastoid bone surface (mm)^*^	L	12.1	11.4	11.6	11.4	11.4	11.7	11.1	11.5	
		R	12.5	12.3	10.9	11.5	11.5	11.7	11.4	11.7	11.6
**Internal acoustic canal**	Volume (mm^3^)	L	5.8	5.4	5.1	4.5	4.2	4.2	5.1	4.9	
		R	5.7	4.7	4.9	4.5	4.4	3.8	5.6	4.8	4.9
Central portion	Length (mm)	L	3.5	2.5	3.5	3.4	2.7	2.8	2.8	3.0	
		R	2.9	2.6	3.3	2.8	2.8	2.6	3.2	2.9	2.9
	CSA (mm^2^)	L	3.5	2.6	2.6	4.0	2.8	3.7	3.0	3.2	
		R	5.9	2.6	2.4	4.5	2.6	3.5	2.9	3.5	3.3
Roof	Length (mm)	L	2.5	2.1	3.1	3.4	2.0	2.1	3.4	2.6	
		R	2.3	1.4	2.7	3.3	2.4	2.4	3.4	2.6	2.6
	Thickness (mm)	L	1.8	1.4	1.9	0.9	1.6	1.1	1.3	1.4	
		R	1.5	1.6	2.3	1.3	1.8	1.2	1.5	1.6	1.5
**Facial canal of the petrous bone**	Volume (mm^3^)	L	8.1	8.4	7.6	7.3	6.2	6.6	7.1	7.3	
		R	8.8	8.5	7.1	6.8	6.7	5.4	6.9	7.2	7.2
Center	CSA (mm^2^)	L	4.1	4.8	5.4	6.3	5.7	4.5	6.1	5.3	
		R	7.5	5.2	5.6	6.0	6.1	4.1	5.9	5.8	5.5
Lateral border	CSA (mm^2^)	L	2.6	2.9	2.0	0.9	0.7	2.5	1.2	1.8	
		R	6.2	1.6	1.8	0.9	0.9	1.5	0.7	1.9	1.9

*Distances (mm), cross-sectional areas (mm^2^) and volumes (mm^3^) were calculated from the acquired tomograms based on simple thresholding and manual segmentation. Spatial measurement of the internal acoustic porus and relevant projections to the bony surface for the extradural middle cranial fossa approach and the retrosigmoid trans-meatal approach, the internal acoustic canal and the facial canal of the petrous bone (Roethlisberger et al., 2015)*.

*VDDS, Vertical distance to the interparietal dural surface; VBDS, Vertical distance to the interparietal bone surface, CSA, Cross-sectional area;*

* *distance from the internal acoustic porus to the interparietal bony [VDBS] and dural surface [VDDS] or the occipital (retromastoid or retrosigmoid) bony surface*.

### Anatomical Assessment

**Figure 4:**
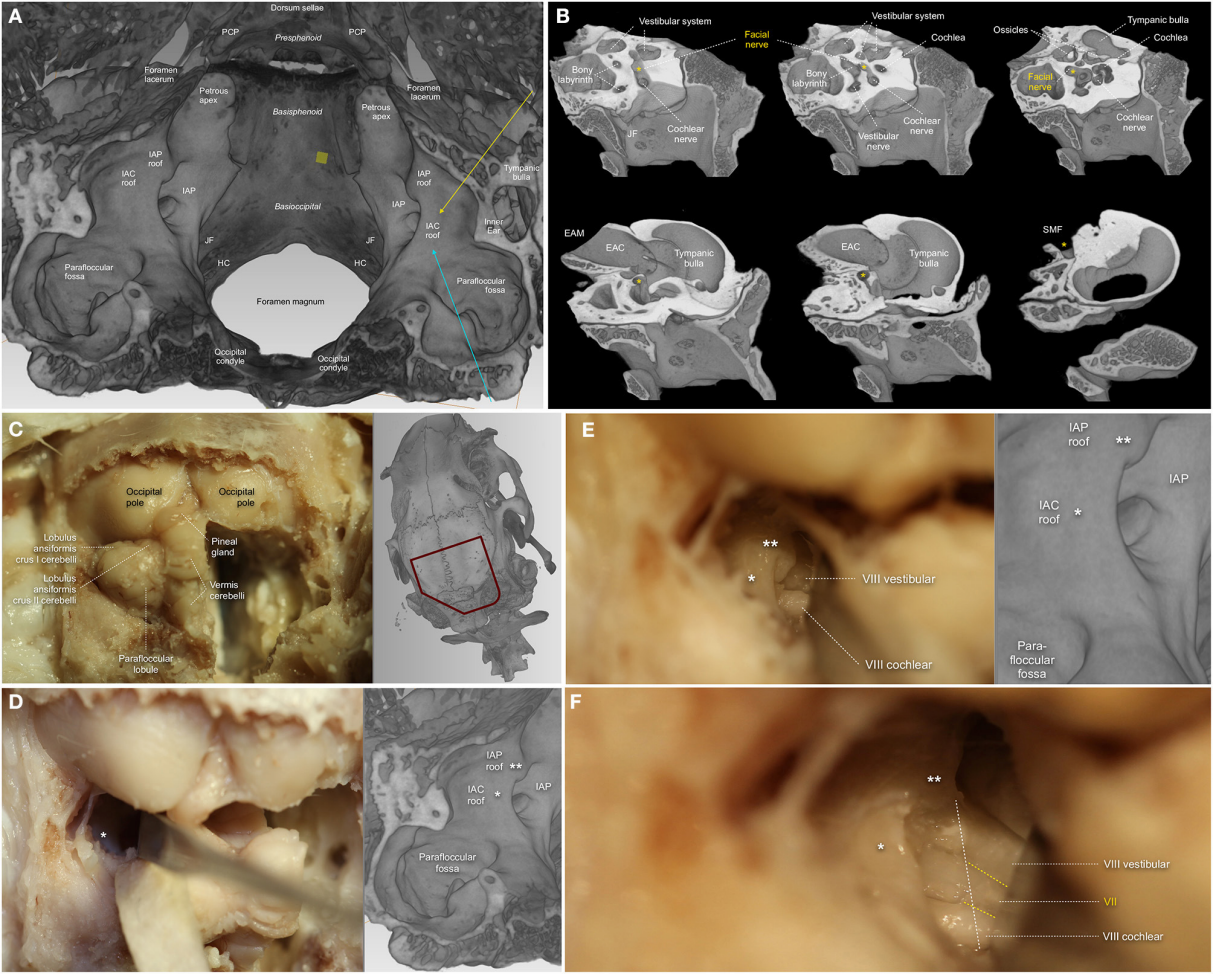
High resolution computed tomography (HRCT) based three-dimensional reconstruction using the nanotom^®^m system and anatomical dissection of a perfusion fixed New Zealand rabbit skull base via the retrosigmoid trans-meatal approach **(A)** The three-dimensional rendering was used to assess details of the adult rabbit skull base. Topographical posterior to anterior view of the lateral (petrous), basioccipital and basisphenoid skull base in a three-dimensional section. The cerebellar floccular and parafloccular lobes are embedded within fossae of the periotic bone contributing to the skull base. The parafloccular fossa is in continuation with the roof of the internal acoustic canal [IAC] and the roof of the internal acoustic porus [IAP]. The middle cranial fossa approach [yellow arrow] and the retrosigmoid trans-meatal approach [blue arrow] use anatomical landmarks to reach the roof of the IAC and the IAP. **(B)** Three-dimensional sections through of the left petrous bone demonstrating the different segments of the facial nerve: the 1st segment (cisternal) runs in company with the vestibulo-cochlear nerve from the lateral side of the trapezoid body in close relationship with the floccular lobule through the CPA to the IAP. Following the 2nd segment (meatal), the facial canal of the petrous bone [yellow *], averagely 11–12 mm in length with an average diameter of 1.2–1.3 mm, harbors the 3rd facial nerve segment (labyrinthine). The facial canal is directed lateralward between the cochlea [C] and the labyrinth toward the geniculate, where the geniculate ganglion is located. The 4th segment (tympanic), defined as the backward projection after the geniculate ganglion to the pyramidal eminence of the nerve. The 5th segment (mastoid) finally courses vertical from the pyramidal eminence to the stylomastoid foramen [SMF], which is bordered by the mastoid, tympanic, and petrous portions of the temporal bone, postero-superiorly to the external auditory canal [EAM] and canal [EAC]. The 6th segment (extratemporal) leaves the canal through the SMF to form the terminal part of the facial nerve before its division in the parotid gland. PCP: posterior clinoid process; JF: jugular foramen; HC: hypoglossal canal. **(C)** The occipital and suboccipital bone has been removed for better visualization of the supra- and infratentorial anatomy. The cerebellum is well-developed and has a queer stalked structure, the parafloccular lobule, on the lateral side of each hemisphere. Three-dimensional rendered images of the same animal based on high-resolution computed tomography (CT) imaging (nanotom^®^m) are provided aside to correlate the microscopic view with the bony landmarks of the lateral skull base in this animal model. **(D,E)** Anatomical concept for a retrosigmoid trans-meatal approach medial to the sigmoid sinus [SS]. Spatular retraction of the parafloccular lobule out of the bony parafloccular fossa, which lies in continuation with the IAC, the IAC roof [white *], the IAP and the IAP roof [white **], exposes the cerebellopontine angle. By pure retraction, the cochlear nerve [VIII, cochlear], located posteriorly in the nerve bundle, and the vestibular nerve [VIII vestibular], located anterior superior within the nerve bundle, can be identified between the IAP and the brainstem for a distance of about 5 mm. **(F)** After microsurgical bony removal of the IAP and parts of the IAC-roof [white line] using a micro high-speed pneumatic diamond-burr, the extra- and intracanalicular nerve bundle is visible. The cochlear nerve [VIII, cochlear] has been slightly dissected and mobilized posteriorly, the vestibular nerve [VIII vestibular] slightly anteriorly, to expose the facial nerve [VII], located anterior inferior within the nerve bundle (Roethlisberger et al., 2015).

After radiological assessment, the perfusion-fixed rabbit skulls were anatomically dissected in the area of the periotic bone and IAP in order to expose the FN in the CPA via a RSM approach. Three-dimensional rendering of the images was helpful to identify the parafloccular fossa as a landmark lying in continuation with the IAC roof and the IAP (Figures 4A,B). As previously described in the literature, the correlation with anatomical dissections of the perfusion-fixed rabbits confirmed the topographical anatomy of a RSM approach in rabbits (Figure 4C; Maurer and Mika, 1983; Lumenta et al., 1988; Levine et al., 1993). A lateral to medial retraction of the parafloccular lobule (Figure 4D), a well-developed anatomical structure in rabbits, exposed the roof of the IAC and the cisternal portion of the FN within the CPA (Lumenta et al., 1988; Levine et al., 1993). In accordance with the detected measurements, microsurgical unroofing of the IAP and IAC was possible (Figure 4E) in order to expose the intracanalicular FN portion after dissection of the cochlear and the superior vestibular nerve (Figure 4F; Roethlisberger et al., 2015).

## Future Research Perspectives for Facial Nerve Repair and Regeneration After CPA-tumor surgery

Current gold standard for treating severed peripheral nerves resulting in a long nerve gap is autologous nerve grafting, although this procedure is associated with several drawbacks including the need for secondary surgery, loss of donor function, limited graft availability and sensory/motor modality mismatch (Tajdaran et al., 2019). With respect to the latter, sensory sural nerve is the most commonly used autograft in humans, which hampers motor axonal regeneration. Nerve allografts were also employed to overcome the difficulties associated with autografts. However, allograft transplantation is generally impaired by host immune rejection (Yu et al., 2020). In this context, proper understanding of the mechanisms underlying nerve injury and repair are of utmost importance. Considering the significance of translational research, *in vitro* and *in vivo* studies mimicking the pathophysiological circumstances of human intracranial FN injuries are therefore highly warranted.

### Biomaterials Based Approaches to Enhance Facial Nerve Regeneration

Poor functional recovery and inevitable limitations associated with nerve grafting in peripheral nerves led to the development of artificial nerve conduits (NC) (Tajdaran et al., 2019) for treating nerve injuries. Failure of nerve regeneration is based on several aspects such as lack of guidance structures, supportive extracellular matrix, neuronal growth factors and Schwann cells. Moreover, biodegradation and tissue growth compatibility of NC are additional key aspects to be considered (Yu et al., 2020). In 1987, Fisch et al. (1987) reported the use of implanted collagen splints for bridging the transected FN after CPA-tumor surgery in humans. The obtained data revealed favorable outcome comparable to peripheral FN anastomosis or grafting. Furthermore, a detailed study using a cat model showed no adverse reaction in response to collagen splint implantation. The regenerated nerve tissue exhibited axonal densities of 50–75% when compared to healthy nerves. Only minimal intraneural fibrosis was observed and no constriction within the collagen was evident (Tables 1, 6). In the sutureless anastomosed FN, the nerves were irregularly shaped due to endoneurial connective tissue ingrowth, indicating the role of collagen tubes for protection and guidance of axonal outgrowth (Fisch et al., 1987). Glasby et al. (1995) reported beneficial effects of autologous muscle graft reinforced with collagen in a sheep model. Sutureless anastomosis of transected intracranial FN resulted in anatomical and functional recovery as evidenced by electrophysiological measurements. Notably, these results are comparable to the outcomes resulting from other peripheral nerve models. Thus, the biomaterial-based approaches hold great promise for repair and regeneration of FN within the CPA. However, the current systematic review demonstrates that experimental evidence remains scarce, with only two (Fisch et al., 1987; Glasby et al., 1995) out of six (Fisch et al., 1987; Glasby et al., 1995; Mattsson et al., 1999; Burgette et al., 2012; Amine et al., 2014; Bendella et al., 2016) studies that ever used a biomaterial-based approach for intracranial FN injury available.

### Bioengineered Scaffolds for Intracranial Facial Nerve Regeneration

Efficient regeneration of severed nerves requires neuronal specific growth factors, permissive microenvironment (i.e., extracellular matrix), axonal adhesion molecules, and guidance structures at a cellular level. Diffusible and substrate-bound factors support axonal guidance and path finding regulated by attractive and repulsive signals. A number of families of signaling molecules were identified, many of which have either an attractant or repellent function (Tear, 1998). Surface structures such as nano- and micro-fibers provide contact guidance for regenerating axons (Manoukian et al., 2020); Neurotrophic factors (NTFs) such as glial cell-line derived neurotrophic factor (GDNF) (Manoukian et al., 2020), nerve growth factor (NGF) and brain derived neurotrophic factors (BDNF) support various sub-populations of motor and sensory neurons, with synergistic effect on axonal branching and elongation (Verderio et al., 2006; Carvalho et al., 2019b). In the presence of GDNF and NGF, longitudinally aligned nanofibers led to directional axonal outgrowth in experimental studies (Carvalho et al., 2019b). Wide ranges of NTFs are readily released from Schwann cells, which play a crucial role for the development and plasticity of the nervous system (Carvalho et al., 2019a). Schwann cells are naturally an ideal choice for therapeutic treatment of nerve injuries. However, Schwann cells isolation and cultures pose serious limitations. Thus, stem cell therapy emerged. Within this context, adipose stem cells (ASCs), bone marrow derived stem cells (BMSCs), umbilical cord derived stem cells (UCSCs), and neural crest derived stem cells (NCSCs) proved to be beneficial for nerve regeneration. Among other cell types, ASCs appear to be more practical given their ease of access in good quantities. The future directions should include the bioengineering of the NC with 3D-guidance structures and stem cells expressing the multiple growth factors for addressing the complex requirements of the severed nerve injuries.

### Experimental Animal Model for Intracranial Facial Nerve Lesions

The working angle in the CPA is quite limited in a rabbit model as previously mentioned. Beside a proper head fixation, even when working under microscopic magnification, the amount of required maneuverability depends on the FN-reconstruction techniques used. The smallest animal reported in the current review, where cranial nerves of the CPA were manipulated, was the rat (Table 7; Mattsson et al., 1999; Burgette et al., 2012; Amine et al., 2014; Bendella et al., 2016; Bonne et al., 2016; Dinh et al., 2018; Chen et al., 2019), followed by the New Zealand rabbit or guinea pig (Table 8; Maurer and Mika, 1983; Lumenta et al., 1988; Levine et al., 1993; Widick et al., 1994; Braun and Richter, 1996; Telischi et al., 1999), cats, dogs and monkeys (Table 9; Chinn and Miller, 1975; Mangham and Miller, 1979; Sekiya et al., 1985, 1990; Fisch et al., 1987; Sekiya and Moller, 1988; Lusk et al., 1990; Greiman and Lusk, 1991; Glasby et al., 1995). However, the rat model published by Amine et al. (2014) and Burgette et al. (2012) for intracranial FN injury required extensive petrous bone drilling, FN rerouting and removal of the cochlear structures to sufficiently expose the FN, on the other hand efficiently creating a post-operative intracranial FN palsy (Table 7). In 2016, Bendella et al. (2016) described the RSM approach in rats using cerebellar retraction to directly manipulate the intra-arachnoidal FN. The authors were able to apply controlled stretch and crush injuries without extensive removal of petrous bone or cerebellar aspiration, thus, including rats besides rabbits as potential rodent models for future studies. However, sharp FN injuries resulting in neurotmesis, crush FN injuries leading to neurapraxia or axonotmesis, or even thermic or ischemic lesions, might be easier to apply in a rabbit model. As initially described by Braun and Richter (1996), the FN in a rabbit model can be exposed inside the CPA within a length of ~ 5 mm by only retracting the parafloccular lobule, while microsurgical unroofing of the IAC gains another 2–3 mm (Figure 4, Table 10; Roethlisberger et al., 2015). Minimal invasiveness and gentle cerebellar retraction without aspiration are important to respect in order to establish a reliable functional animal model. Reports are given that lesioning the ipsilateral anterior lobe of the cerebellar cortex in rabbits is associated with severe deficits in conditioned response extinction, e.g. the eyelid response (Perrett and Mauk, 1995). Further on there is evidence that defects created in the midline cerebellum in rats showed difficulties in maintaining equilibrium and tended to be slower than those with lesions laterally, therefore affecting motor function. Rats with lateral lesions demonstrated deficits in spatial orientation, whereas midline lesioning caused deficits in visuomotor coordination (Joyal et al., 1996).

As extracted from the reviewed human studies, the different types of intracranial FN injuries after CPA-tumor surgery should be systematically addressed in future experiments. Sharp (neurotmesis) injuries have been produced using microsurgical diamond knifes (Glasby et al., 1995), crush injuries (neurapraxia or axonotmesis) mediated by a jewelers forceps (Burgette et al., 2012), thermic lesions by application of heated water (Braun and Richter, 1996) or high speed drilling (Burgette et al., 2012), ischemic lesions by selectively ligating the internal auditory artery, the petrosal branch of the middle meningeal artery (petrosal artery) and/or the stylomastoid artery (Takeda et al., 2008, 2015). To bridge the gap from this context to *in vivo* studies, New Zealand White rabbits might be a very cost-effective and valuable option nowadays in simulating nerve regeneration after intracranial FN injury. Additionally, they can provide an important translational insight from *in vivo* series to clinics (Sherif et al., 2011). The current review established the evidence of CPA surgeries and effective manipulation of the FN within the CPA, based on several previous studies (Table 8) and was further confirmed with radiological examinations and anatomical dissections (Figure 4, Table 10; Maurer and Mika, 1983; Lumenta et al., 1988; Levine et al., 1993; Widick et al., 1994; Braun and Richter, 1996; Telischi et al., 1999; Roethlisberger et al., 2015). It is of note that a balanced anesthesiologic approach should be the gold standard in systematically performing experiments in this area, besides an established surgical protocol, to avoid confounders (Wanderer et al., 2020). Analgesia should allow for placing a metallic head-post-device firmly attached to the vertex, so that the animal's head can be securely fixed. In addition, core body temperature should be maintained at 38°C with a thermoregulated heating blanket coupled to a rectal thermometer. With a balanced anesthesiologic protocol, operation times of up to 240 min are granted, providing low morbidity and long-term mortality. When using a ketamine/xylazine anesthesia, spontaneous exhibition of vibrissae twitching and corneal reflexes indicate the need for supplemental medication to maintain anesthesia (Widick et al., 1994). Furthermore, the low mortality of this protocol provides the unique possibility of an excellent long-term observation interval considering all the important clinical end-points necessary for a FN injury model. Moreover, facial deficits and regeneration processes can easily be objectified using the Rabbit Grimace Scale, blink reflex, whisker movements, and electrophysiological and histological examinations, as extensively described in the literature (Keating et al., 2012). Standardized pre-operative assessment of each rabbit is performed by using the ASA-classification (Irlbeck et al., 2017). Because of the well-feasible long-term follow-up in New Zealand White rabbits, a time-dependent delivery of different growth factors derived from semi-liquid or fibrillar web-like biomaterials could be examined. Additionally, access to the CSF system in rabbits is easily given by applying a puncture needle directly into the cisterna magna. Techniques for this approach have been described elsewhere (Croci et al., 2019). With a fixing system, as well with implantation of a subcutaneous minipump, a continuous and dose dependent application of medication, even neural growth factors, might be feasible (Croci et al., 2019).

### Strategies for Direct End-to-End Facial Nerve Anastomosis

**Figure 5:**
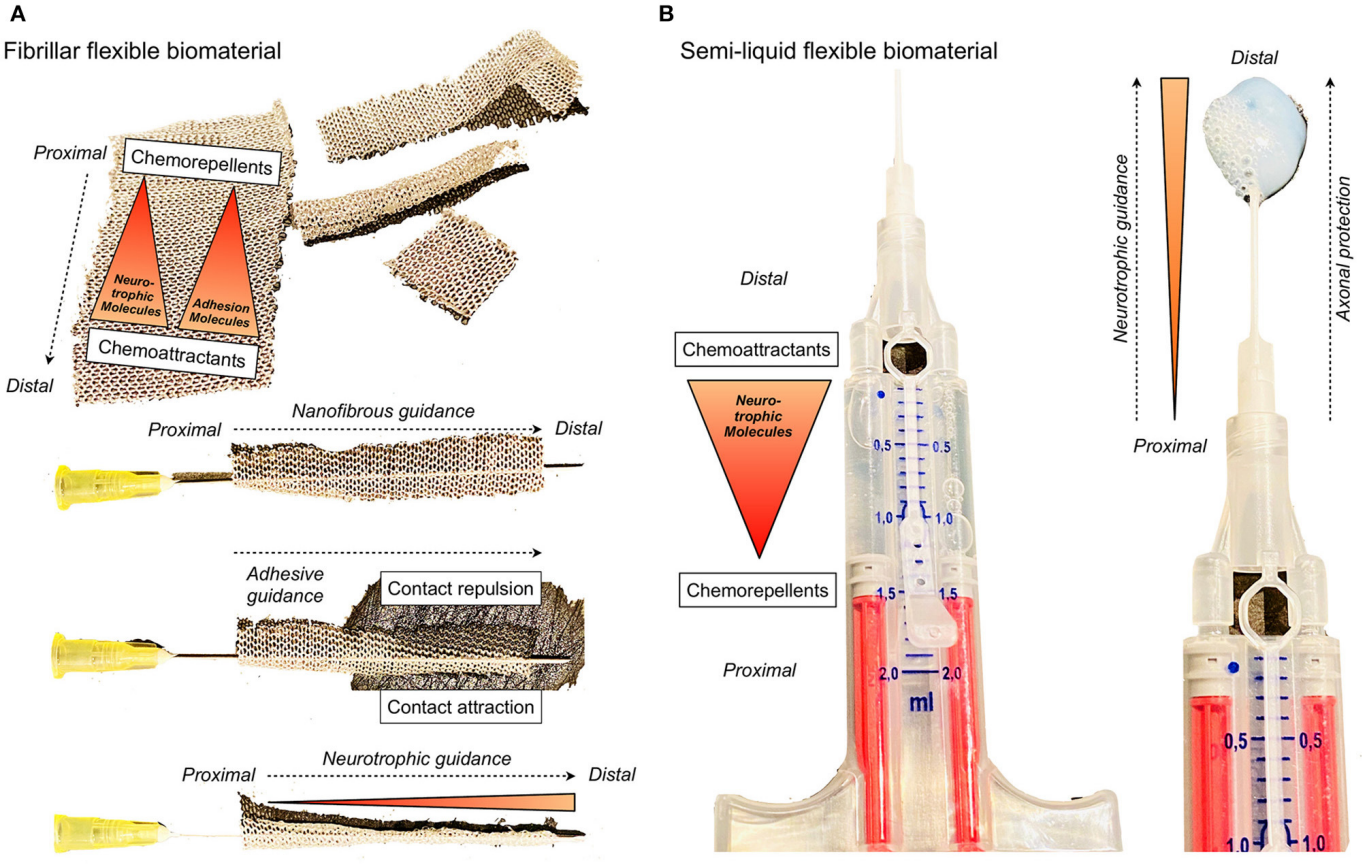
Common biomaterials that are used in skull base neurosurgery and for intracranial facial nerve reconstruction: **(A)** Fibrillar hemostyptical material is flexible and develops adhesive characteristics in contact with water or blood. Such a biomaterial could be enhanced with nanofibrous structures, adhesion molecules and neurotrophic factors to establish directional axonal guidance for neuronal regeneration after intracranial facial nerve crush injury. **(B)** Fibrin glue is a surgical formulation containing separately packaged human fibrinogen and human thrombin in two vials, which are mixed immediately before application, containing other factors of hemostasis. Such a semi-liquid medium could be enhanced with adhesion molecules and neurotrophic factors to establish directional axonal guidance, and additionally act as protective layer for neuronal regeneration after intracranial facial nerve crush injury.

Based on the current review of human studies, the first discussed FN reconstruction technique after intracranial FN injury is direct end-to-end FN anastomosis with micro-suture of the endoneurial ends within the CPA (Bacciu et al., 2009). If regenerative strategies have to be tested in an experimental rodent model, cutting the nerve via axonotmesis or neuronotmesis and direct coaptation with an end-to-end anastomosis might be difficult. The use of fibrin glue to oppose the nerve ends without interrupting the nerve gap has been proposed, and became common practice in the clinical environment (Ramos et al., 2015). Fibrin glue is a surgical formulation containing separately packed human fibrinogen and human thrombin in two vials, which are mixed immediately before application, containing other factors of hemostasis (Figure 5A; Atrah, 1994). Such constructs are usually stabilized using fibrillar hemostatic materials containing oxidized regenerated cellulose (Figure 5B). Similar semi-liquid or fibrillar flexible web-like biomaterials could augment FN regeneration in the CPA. The establishment might be feasible by using a hydrogel-based drug delivery system, in terms of a “modified fibrin glue,” representing a valuable option for direct macromolecule delivery to the site of the surgical repair of the injured nerve, as already described by Tajdaran et al. (2019). Guided axonal growth might be an interesting topic of research, directed from the brain stem root-entry zone nerve stump to the peripheral intra-meatal nerve stump, stabilized by nerve wrapping with a “modified flexible and fibrillar web” employing the integration of a proximal-to-distal directional gradient of excreted growth factors (Figure 5). Based on their molecular properties, hydrogel and nerve wraps are very well-suitable for implantation at several different anatomical locations, as in a narrowed area like the CP angle of rabbits. Furthermore, the biocompatibility and -degradability of delivery systems like these eliminates the need for a second surgery removing all implanted material. Given the possibility of wrapping nerve stumps or using hydrogel-based materials, even sharp FN injuries via axonotmesis or neuronotmesis could be repaired and tested in a narrow space like the rabbit CPA.

### Strategies for Cable Nerve Grafting

Commonly used tubes and tubular scaffolds as conduits prolong the working angle for direct nerve suturing and are thus not easily implanted in anatomically limited areas such as the rabbit CPA. The lack of sufficient space might be an inacceptable limitation and such experiments might thus only be feasible in in larger non-rodent models like cats or sheep. For the case of these animal models, there is limited data evidence for the feasibility of experiments relying on sectioning the FN in the CPA and testing reanastomosis using collagen splints or short freeze-thawed muscle autografts secured in place with Tisseel fibrin glue (Table 9). As previously mentioned, both non-rodent animal models demonstrated reliable FN palsy rates after application of an intracranial FN lesion along with acceptable long-term FN recovery rates. Hence, these models promise good reproducibility that is needed to test adjunctive biomaterials (Fisch et al., 1987; Glasby et al., 1995). As such, acellular nerve allografts with guided axonal growth and exogenous factor release might be an excellent approach to improve nerve regeneration in long nerve gap injuries (Figure 5). However, large animal models such as cats, sheep and monkeys, have organizational, ethical and infrastructural drawbacks nowadays. Reasons are that the availability of corresponding infrastructures with a dedicated pre-, peri-, and post-operative setting is limited. Statutory restrictions are very high, as well as animal costs (animal shipping, acclimatization period, housing, anesthesia, post-operative surveillance, medications) and costs per person (e.g., surgeon, veterinarian and anesthetist). However, the rabbit model, considering cost-effectiveness and feasibility, might be well-suited for research purposes addressed in this manuscript. Nevertheless, in terms of ethics, the debate about which animals to use for a specific research question is still ongoing. Independent of the chosen animal, the degree of severity of the procedures performed has clearly to be stated in the ethics approval. There might be a clear tendency to use larger mammals to reproduce transcranial approaches and intracranial FN palsies, however, the invasiveness of the procedures might limit such experiments from an ethical standpoint. This is well-reflected within our review, where larger mammals were part of experiments in earlier studies, and newer studies tend to use small and larger rodents (Tables 7–9). All animal experiments, independent of species, have to be planned and conducted in accordance to the ARRIVE (Animal Research: Reporting of *in vivo* Experiments) guidelines. Regarding a broad-based acceptability, animal models should be standardized, easy reproducible, cost-effective and associated with a low mortality and morbidity, independent from above mentioned ethical aspects (Percie du Sert et al., 2020).

## Limitations

Unfortunately, many studies do report on post-operative FN function. However, they either report only one post-operative state, making it impossible to see how many patients improved, or they only report the immediate postoperative state not following them up, again hindering the possibility to assess improvement of FN function. This matter should be evaluated in the future in a systematic way in order to make more accurate predictions on FN function outcome depending on treatment, extent of resection and other factors. We included studies where HB I and II were combined. This can lead to selection bias, underestimation of FN impairment and overestimation of FN recovery. Analysis of follow-up time was not possible as it was either not reported or differed significantly with a range of weeks to years among the included publications. Ideally, a large prospectively conducted multi-center study using clear reporting guidelines will enable any reviewer or reader to reliably reproduce the data.

## Conclusion

Experimental crush injury models of the intracranial FN may be able to more accurately reflect the common FN injury patterns after human CPA-tumor surgery. It is for this reason that these models should be considered as distinct pathophysiological entities apart from peripheral and/or transection injuries. This proposition is supported by scarce experimental evidence on injuries of the intra-arachnoidal FN that lead to a higher neuronal cell death in the facial motor nucleus and thus a poor clinical outcome. Rats and New Zealand rabbits allow for sufficient exposure and manipulation of the intracranial FN. Future experimental approaches for intracranial FN regeneration should focus on bioengineered, flexible and semiliquid biomaterials, which are commonly used in skull base surgery, endowed with topographical guidance structures, stem cells and neurotrophic factors, for addressing the various needs of different subsets of the neurons and for regulating the axonal regeneration and path finding.

## Data Availability Statement

The original contributions presented in the study are included in the article/supplementary materials. Further inquiries can be directed to the corresponding author/s.

## Author Contributions

MR conceived the presented idea. MR, DK, RG, and SMad developed the concept. ICH and MR verified the analytical methods. DK, RG, LM, SMad, and SMar encouraged MR and EN to investigate the animal model and perfusion fixation, MR to perform anatomical dissections, CB to perform the high resolution computed tomography examinations, and ICH as well as MR to perform the systematic literature review. ICH, NJ, SW, CB, EN, RK, VW, DK, LM, RG, SMad, and MR contributed to the interpretation of the clinical and pre-clinical results and wrote significant parts of the manuscript. MR and SMad supervised the findings of this work. MR took the lead in writing the manuscript. All authors provided critical feedback and helped shape the research, analysis, and manuscript.

## Conflict of Interest

The authors declare that the research was conducted in the absence of any commercial or financial relationships that could be construed as a potential conflict of interest.

## Abbreviations

CPA: cerebellopontine angle
FN: facial nerve
IAP: internal acoustic porus
IAC: internal acoustic canal
CSF: cerebrospinal fluid
VS: Vestibular schwannoma
RSM: retrosigmoid-transmeatal
TRL: trans-labyrinthine
MCF: middle cranial fossa.
